# Hedgehog pathway activity downstream of Smoothened is regulated specifically by basal ciliary PKA

**DOI:** 10.1186/s11658-026-00915-x

**Published:** 2026-04-09

**Authors:** Hongyu Zhang, Shujing Chen, Zhuoya Huang, Jin Ben, Yanling Wei, Guangxin Chen, Changxin Wu, Haibo Xie, Philip W. Ingham, Zhonghua Zhao

**Affiliations:** 1 Biomedical and Health Laboratory in Shanxi Province, Institute of Biomedical Sciences, The Key Laboratory of Chemical Biology and Molecular Engineering of Ministry of Education, School of Life Science, Shanxi University, Taiyuan, 030006 China; 2 School of Life Science, Yunnan University, Kunming, 650504 China; 3 Department of Life Sciences, University of Bath, Bath, BA2 7AY UK; 4 College of Marine Sciences, Ocean University of China, Qingdao, 266000 China

**Keywords:** HH pathway, Primary cilia, Cilia targeted PKA, Nphp3N-AKAR2, Smoothened

## Abstract

**Background:**

Effectors of the vertebrate Hedgehog (HH) signaling pathway are organized within primary cilia (PC). Protein kinase A (PKA), a ubiquitously distributed kinase in most cells, functions as a specific negative regulator of the HH pathway. Its functional specificity in the HH pathway has been suggested to be controlled by cyclic adenosine monophosphate (cAMP) in PC. However, the regulation of PKA and its roles in PC remain unclear, partly owing to the lack of observations regarding PKA localization in PC during the resting state of HH signaling, as well as conflicting reports on the dynamic changes in ciliary cAMP levels and HH pathway activity. Here, we clarify that PKA with basal activity in PC specifically regulates the HH pathway and confirm that Smoothened (SMO)-mediated HH pathway activation may not be fully dependent on its inhibition of ciliary PKA activity.

**Methods:**

To investigate the role of PKA during HH pathway, we have developed an improved ciliary-localized Förster resonance energy transfer (FRET)-based A-kinase activity probe (Nphp3N-AKAR2-CR) for real-time monitoring of ciliary PKA activity in both cultured cells and living embryos. Additionally, by leveraging a highly efficient ciliary targeting peptide (Nphp3N), we specifically delivered PKA variants to either PC or cytoplasm, thereby dissecting the regulatory roles of PKA in the HH pathway across different subcellular compartments. Furthermore, we performed constitutively active SMO variant (SMOA1)–forskolin (FSK) titration assays to validate the dose-dependent relationship underlying SMO-mediated inhibition of PKA.

**Results:**

Basal ciliary PKA activity in cells was detected by this probe, despite the absence of observable PKA catalytic subunits in PC. Only ciliary-targeted PKA can modulate the HH pathway, even when PC integrity is disrupted. Notably, ciliary PKA activity is barely changed by either inhibition or activation of the HH pathway at the level of SMO. Furthermore, even low concentrations of FSK efficiently inhibit the HH pathway in the presence of SMOA1.

**Conclusions:**

A basal level of PKA localized in PC but not cytoplasm specifically regulates HH signal transduction, and SMO-mediated activation of the HH pathway may not be solely attributed to the direct regulation of ciliary PKA activity.

**Graphical abstract:**

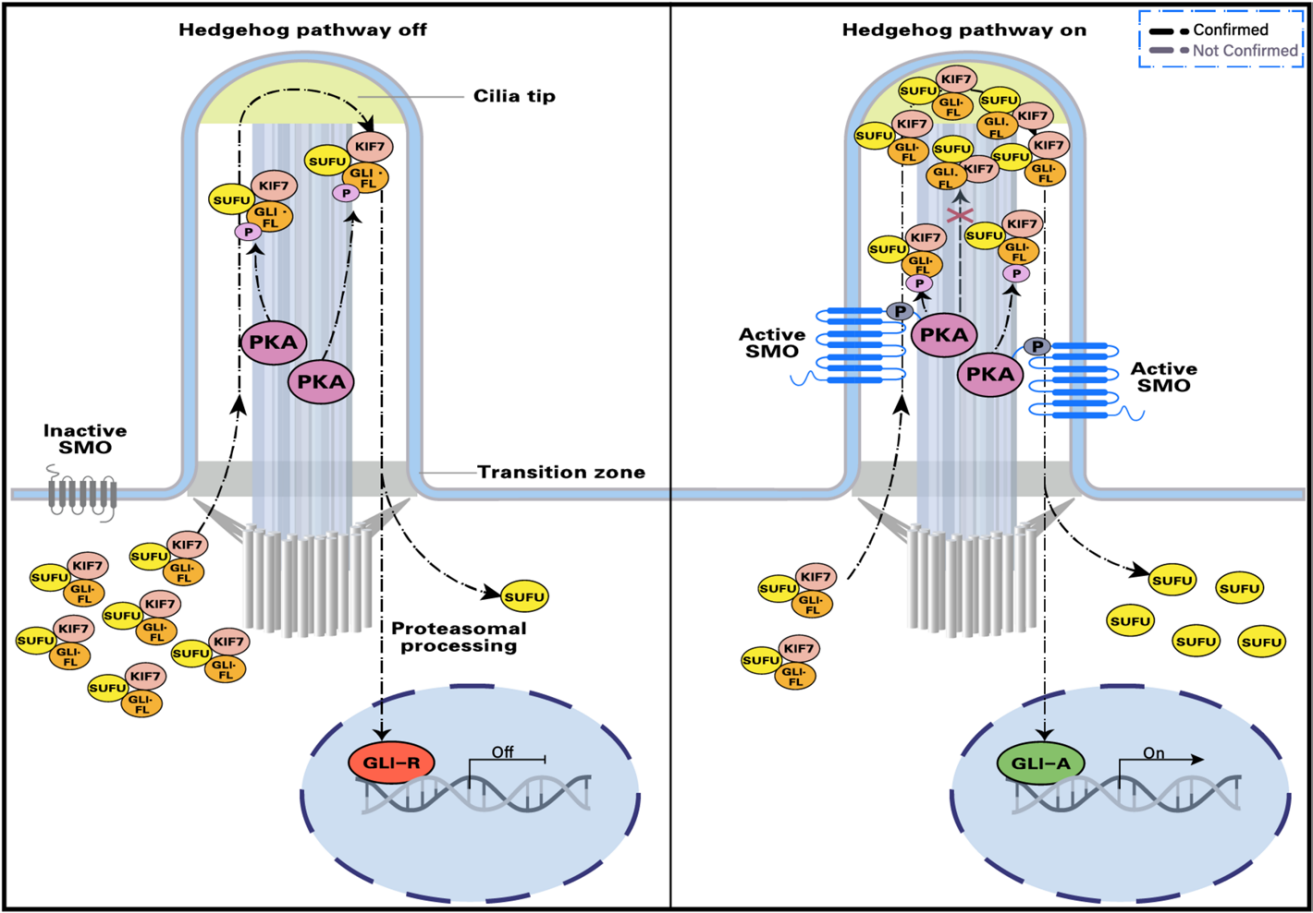

**Supplementary Information:**

The online version contains supplementary material available at 10.1186/s11658-026-00915-x.

## Background

The HH signaling pathway is conserved from cnidaria to vertebrates, playing critical roles in embryonic development, adult tissue, and metabolic homeostasis [1]. Abnormal HH pathway activity results in multiple developmental defects and is also implicated in the onset and progression of various malignancies [2–4]. The mechanism of HH signal transduction has been extensively studied in vertebrate systems, including mice, zebrafish, and cultured cells, as well as invertebrates, especially *Drosophila*, where it was first characterized [1]. Most core components of the pathway are shared between phyla and have highly similar mechanisms controlling HH signal transduction. However, this process is more complex in vertebrates, primarily owing to its dependence on the PC, a highly specialized subcellular compartment.

A major event in the regulation of HH pathway activity by PC is the divergent phosphorylation of glioma-associated oncogene (GLI) proteins, which generates either the full-length active form or the truncated repressor form [5, 6]. As one of the key negative regulators of the HH pathway, PKA is considered to be the critical kinase that phosphorylates GLI and promotes its cleavage into a repressor form, thereby inhibiting the activation of HH pathway target gene [7–15].

The PKA holoenzyme is a heterotetramer composed of two catalytic subunits (PKA-C) and two regulatory subunits (PKA-R). These subunits are ubiquitously expressed and involved in many intracellular signaling pathways that mediate numerous physiological processes, including cell proliferation, metabolism, memory formation, and muscle function [16–18]. The localization of PKA to specific subcellular compartments is crucial for its diverse signaling functions [19]. PKA activity is primarily controlled by cyclic adenosine monophosphate (cAMP), which binds directly to the PKA-R subunit and thereby releases active PKA-C [20]. Generally, cAMP levels are dependent upon adenylyl cyclases (ACs), which are activated or inhibited by Gα_s_ or Gα_i_ subunits, respectively, in response to G-protein-coupled receptor (GPCR) stimulation. Furthermore, heat-stable protein kinase inhibitor (PKI) proteins can also modulate PKA activity through direct interaction with the catalytic subunit of PKA [21]. In the HH pathway, SMO, an F-family GPCR, has been hypothesized to inhibit PKA activity, either directly by sequestering the PKA-C via a pseudosubstrate domain within its intracellular C-terminal domain [22, 23] or indirectly by activating Gα_i_ [24–26]. GPR161, a typical ciliary GPCR, has also been implicated in HH pathway regulation by increasing PKA activity through Gα_s_ in the ventral neural tube, chondrocytes, and other ciliated cell types [27, 28]. Both GPCRs localize to PC and show opposing ciliary accumulation in response to HH signaling, consistent with the regulation of HH pathway activity by PKA occurring within the PC. In line with this, PKA-R subunit has been directly visualized in PC [29]. By contrast, although ciliary localization of PKA-C subunit has been inferred from proteomic analysis [29], it has only been visualized in PC following HH pathway activation, coincident with SMO translocation [23]. Such distribution dynamics seem paradoxical if HH pathway activity is supposed to be suppressed by PKA within the PC. Moreover, the prevailing model that PC maintain relatively higher cAMP levels than the cytoplasm in the HH-OFF state, followed by a reduction in cAMP upon HH activation, remains controversial, further obscuring the association between ciliary PKA and HH pathway activity [30, 31].

Previous studies have used the 5HT_6_-AKAR4 to assay PKA activity in PC [30]. By targeting the FRET-based A-kinase activity probe (AKAR2) to the PC using the N-terminal region of zebrafish Nephrocystin-3 (Nphp3N) [32], we have established an improved biosensor that facilitates real-time dynamic monitoring of PKA in the PC of both cultured cells and living embryos. This sensor can detect changes in ciliary PKA activity in response to FSK treatment, as well as to the expression of the dominant negative PKA-R variant and PKI, confirming the presence of basal PKA activity in PC. Furthermore, by utilizing the Nphp3N to specifically deliver different PKA variants to either PC or the cytoplasm in both cells and embryos, we found that only ciliary-targeted PKA specifically regulates the HH pathway, even in the absence of intact PC. Unexpectedly, perturbation of the HH pathway at the level of SMO did not change PKA activity within PC. Moreover, constitutive activation of SMO was insufficient to overcome the effects of increased PKA activation mediated by a range of FSK concentrations, which contradicts the expectation that SMO inhibits PKA by directly sequestering it in PC. Taken together, our findings suggest that a basal level of PKA localized in PC but not in the cytoplasm specifically regulates HH signal transduction in cells.

## Methods

### Cell culture of HEK293T and NIH3T3 and transfection

HEK293T and NIH3T3 cells, procured from the American Type Culture Collection (ATCC), were maintained in Dulbecco’s modified Eagle medium (DMEM) supplemented with 10% fetal bovine serum (FBS) and 10% penicillin–streptomycin, at 37 °C with 5% CO_2_. For ciliogenesis, the cells were initially rinsed with phosphate-buffered saline (PBS) and then subjected to serum starvation by culturing in FBS-free medium for 24 h. For transfection, Lipofectamine 3000 (Thermo Fisher Scientific, L3000015) or polyethylenimine (PEI; MedChemExpress, HY-K2014) was used according to the manufacturer’s protocol. To activate ACs, 10 μM FSK (MedChemExpress, HY-15371) was added to the seeded cells and stimulated for 30 min. For experiments investigating the effect of 8-Br-cAMP on ciliary PKA activity, 10 μM 8-Br-cAMP (MedChemExpress, HY-12306A) was added to cells expressing Nphp3N-AKAR2-CR, followed by stimulation for 30 min. To induce or inhibit the HH signaling, the cells were plated and treated with either 1 μM SMO agonist (SAG; MedChemExpress, HY-12848) or 5 μM cyclopamine (MedChemExpress, 4449-51-8), respectively, and then subjected to the following serum starvation for 24 h prior to collection. Detailed operating conditions are listed in Supplementary Table S1.

### Zebrafish husbandry

Adult zebrafish of the AB strain, sourced from the China Zebrafish Resource Center, were housed at 28 ℃ on a 14 h light/10 h dark cycle within the zebrafish facility of Shanxi University. Embryos were obtained from natural mating and grew in E3 medium (5 mM NaCl, 0.17 mM KCl, 0.33 mM CaCl_2_·2H_2_O, and 0.33 mM MgSO_4_·7H_2_O). The embryos were staged according to the standard developmental staging guidelines [33]. For experiments measuring ciliary PKA activity under different treatment conditions, FSK was administered to zebrafish embryos at 6 hours post-fertilization (hpf) and maintained at the indicated concentrations until immediately before sample analysis. CyA was used at a final concentration of 40 μM for Hh signaling inhibition in zebrafish embryos.

### DNA constructs, RNA synthesis and injection

The transient expression constructs of *pCS2* + *-nphp3N-AKAR-CR*, *pCS2* + *-cPKA/dnPKA*, *pCS2* + *-nphp3N/(G2A)-cPKA/dnPKA-eGFP*, *pCS2* + *-cPKA/dnPKA-mRAB23(S23N/Q68L), pCS2* + *-IFT88-Flag* and the transgenic construct of *pMiniTol2-β-actin-nphp3N-AKAR-CR* and *pMiniTol2-en2a-eGFP* were generated by Gibson assembly. The primers used for cloning are listed in Supplementary Table S2. The transient expression constructs of *pCS2* + *-SMO-eGFP*, *pSP64-shh* and *pDB600* were used as described [34, 35]. The transient expression constructs *pCS2* + *-Nphp3N-PKIα* were synthesized by Sangon Biotech (Shanghai, China). The vectors *psPAX2* and *pMD2G* were kindly provided by the lab of Jianguo Li (Institute of Biomedical Sciences, Shanxi University, Taiyuan, China). cPKA was a corresponding mutation (H89Q; W198R) in the catalytic subunit of zebrafish PKA as previously described [36]. dnPKA was modified in accordance with previous study [37].

For capped mRNA synthesis, the transient expression constructs were linearized by NotI for *pCS2* + constructs, BamHI for *pSP64-shh* and XbaI for *pDB600* plasmid and then purified by Zymogen DNA concentrator. The mRNA was produced using either Sp6 or T3 Invitrogen Ambion mMessagemMachine^®^ Kit according to the promoter in the constructs following the instructions, then purified mRNA was stocked in −80 ℃ for further use.

For transient expression of the fusion protein in zebrafish embryos, 150 pg of mRNA was injected into one-cell stage embryos.

### Generation of zebrafish transgenic lines

To generate the *sx1002* stable transgenic line, the *pMiniTol2-β-actin-nphp3N-AKAR2-CR* construct was co-injected with Tol2 transposase mRNA into one-cell stage embryos. T_0_ embryos with strong green and red fluorescence were screened at 24 hpf and inoculated as potential founders. Mature T_0_ fish were outcrossed with wild-type, and T_1_ offspring with consistent fluorescence were selected as stable transgenic lines.

To create the stable transgenic line Tg(*en2a:eGFP*)^sx1005^ (*sx1005*), Tol2 transposase mRNA was co-injected into one-cell stage embryos along with the *pMiniTol2-en2a-eGFP* construct [34]. T_0_ embryos with strong green fluorescence were selected at 24 hpf and inoculated as potential founders. T_1_ embryos with steady fluorescence obtained by outcrossing of potential founders with wild type were selected as stable transgenic lines.

### Förster resonance energy transfer (FRET) imaging

FRET assay was performed as described [38]. Fluorescence imaging was performed using an LSM 710 confocal inverted microscope (Zeiss). Time stacks of images (512 × 512 pixels, typical field of view 135 μm × 135 μm, 600 Hz scanning frequency) were acquired with a 63× oil immersion objective at room temperature under atmospheric conditions. The pinhole was set at 1 AU. The Clover was excited at 488 nm, its emission was detected in the range 500–550 nm, and the mRuby emission was detected in the range > 561 nm. Pre-experiments under the emission range of mRuby were carried out to test the bleaching effect. All regions of interest (ROIs) are 90% bleached to maximize the bleaching of the acceptor with little or no effect on the donor. At least two regions were selected for each field, with one nontarget set as the background and the random cell region set as the object. The acquisition of all channel images was set up as a time series of five cycles, with bleaching starting after two cycles.

For each sample, image time series have been acquired by selecting the field of view populated with more than 40 cells. After acquisition, data were analyzed through the FRET package of Zen Imaging Software. The ROIs including each cell were selected and the Clover and mRuby fluorescence intensity at different time points before and after bleaching in both acquisition channels were calculated. The relative FRET efficiency was calculated with the following formula: FRET = 100 × (IF_Donor, after_ − IF _Donor, before_)/IF _Donor, after_. The raw data were then further elaborated with Excel™.

### Whole-mount in situ hybridization

The probes of *ptch2*, *olig2*, and *nkx2.2a* for in situ hybridization were prepared as described [7, 39, 40], and in situ hybridization was performed as described [41]. Images were captured using Zeiss Imager M2 microscope.

### Western blot analysis

Total protein of embryos and culture cells was prepared as described [34, 42]. In brief, the embryos were lysed using lysis buffer (20 mM Tris HCl (pH 7.4), 150 mM NaCl, 1% Triton X-100, 10% glycerol, 2 mM ethylenediaminetetraacetic acid (EDTA), and 1 mM phenylmethylsulfonyl fluoride (PMSF)) at the ratio of 1μL lysis buffer per embryo. The culture cells were lysed using radioimmunoprecipitation assay (RIPA) buffer (50 mM Tris–HCl at pH 7.5, 150 mM NaCl, 1% Triton X-100, 1% sodium deoxycholate, 0.1% sodium dodecyl sulfate (SDS), 1 mM EDTA, and 1 × protease inhibitors). The supernatant was collected after centrifugation at 12,000*g* for 20 min at 4 ℃, and then was mixed with loading buffer (37.5 mM Tris HCl, pH 7.4; 3% SDS; 0.01% bromophenol blue; 6.25% glycerol; and 100 mM dithiothreitol (DTT)) and boiled for 5 min before polyacrylamide gel electrophoresis (PAGE) gel assay. Total protein of 40 embryos was separated on 10% acrylamide gel, and then the proteins were transferred onto the polyvinylidene difluoride (PVDF) membrane (GE Healthcare Life Science, 10,600,023). The blocking and antibody incubation were performed accordingly. A complete list of the antibodies and dilutions employed in this study is provided in Supplementary Table S3. Horseradish peroxidase (HRP)-labeled secondary antibodies were detected with the super sensitive enhanced chemiluminescence (ECL) substrate kit (Thermo Fisher Scientific, 34,580). And the images of the western blot were captured using GE Healthcare infrared imager.

### Immunofluorescence

Immunofluorescence (IF) staining on embryos was performed as described [43]. In brief, the embryos were fixed with 4% paraformaldehyde (PFA) at room temperature for 2 h and then stored at −20 °C with absolute methanol after dehydration. To proceed IF, the fixed embryos were permeabilized in acetone after being rehydrated. Then the embryos were subsequently incubated by blocking buffer (Dulbecco’s phosphate-buffered saline (DPBS), 1% BSA, 1% dimethyl sulfoxide (DMSO), 0.5% TritonX-100), primary and secondary antibodies in blocking buffer, and then washed in PBTX (DPBS, 0.5% Triton X-100). The washed embryos were stored in 70% glycerol/DPBS at 4 °C. The antibodies and their titration used in this study are listed in Supplementary Table S3.

For cell IF staining, the cells were prepared on 14-mm coverslips (NEST, 801,010) and fixed with 4% PFA. The IF staining was performed as described [44], with the blocking buffer of 1% bovine serum albumin (BSA) in PBS, and antibodies in PBS. The antibodies and their titration used in this study are listed in Supplementary Table S3. The cells on the coverslips were mounted onto microscope slides using the antifade reagent (Beyotime Biotechnology, P0131), after IF staining.

The imaging was conducted using LSM 710 confocal microscope (Zeiss Microsystem, Germany).

### RNA isolation and quantitative RT-PCR

Total RNA was isolated from zebrafish embryos at 24 hpf using TRIzol reagent (Invitrogen). First-strand complementary DNA (cDNA) was synthesized from 1 µg of total RNA using SuperScript IV reverse transcriptase (Invitrogen) with oligo(dT) primers (Sangon Biotech). Quantitative real-time polymerase chain reaction (qRT-PCR) was performed on a BioRad detection system using GoTaq qPCR Master Mix (Promega), with 0.2 µL of cDNA as the template. Gene expression levels were normalized to the housekeeping gene *β-actin*. Primers specific for the target genes (*gli1*, *ptch1*, and *ptch2*) are listed in Supplementary Table S2. All experiments were performed with three biological replicates and three technical replicates per sample.

### Statistics

All experiments were conducted with a minimum of three replicates. All statistical analyses were performed using Prism version 7.0 software. Results are presented as mean ± standard deviation. For experiments with two groups, we performed unpaired two-tailed Student’s *t* tests. For comparisons among three or more independent groups, we have performed one-way analysis of variance (ANOVA) followed by Tukey’s multiple-comparisons test. Statistical significance is indicated in the figures as follows: *p < 0.05, **p < 0.01, ***p < 0.001. All image processing and analysis was performed by using ImageJ (version v1.52i; US National Institutes of Health, Bethesda, MD, USA).

## Results

### Construction of a biosensor that accurately reports cilia-specific PKA activity

**Fig. 1:**
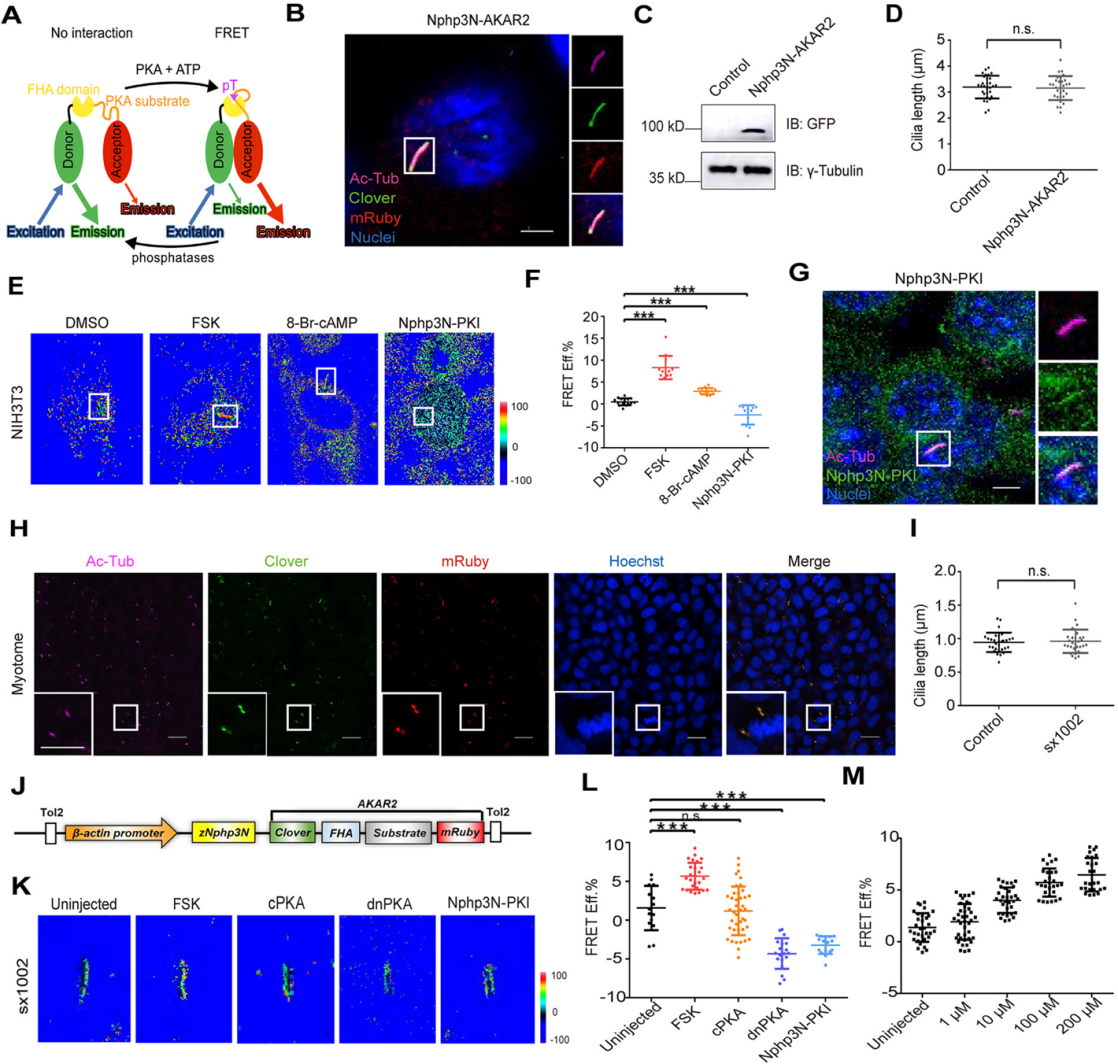
The Nphp3N-AKAR2-CR biosensor can accurately and efficiently report PKA activity in PC. **A** Schematic model of the AKAR2-CR illustrating PKA activity. **B** Specifically ciliary localization of Nphp3N-AKAR2-CR as indicated by colocalization of Clover and mRuby. The cilium labeled by acetylated tubulin (Ac-Tub) and the nuclei were labeled by Hoechst. Scale bars, 5 μm. **C** The expression of Nphp3N-AKAR2-CR was detected using GFP antibody against AKAR2-CR. **D** Cilia length in NIH3T3 cells expressing Nphp3N-AKAR2 is not changed (*n* = 30 cilia). **E** The representative FRET ratio image of ciliary AKAR2-CR in NIH3T3 cells treated with DMSO, 10 μM forskolin (FSK), 10 μM 8-Br-cAMP, or overexpressing Nphp3N-PKI. **F** Statistical analysis of the ciliary FRET efficiency in NIH3T3 cells in response to DMSO, FSK, 8-Br-cAMP, and Nphp3N-PKI. (*n* = 12 cilia). **G** Colocalization images of Nphp3N-PKI within PC in NIH3T3 cells. Cilia were labeled by Ac-Tub. The nuclei were labeled by Hoechst. Scale bars, 5 μm. **H** The AKAR2 specifically localized in PC of the *sx1002* as indicated by Clover and mRuby. Cilia were labeled by Ac-Tub. Each inset on the left corner denotes the enlarged site encircled by white square. The nuclei were labeled by Hoechst. Scale bar, 5 μm. **I** Length of PC in myotome of *sx1002* embryos is the same as that in wild type embryos (*n* = 30 cilia from three embryos). **J** Schematic diagram of the *Nphp3N-AKAR2-CR* vector used for generating ciliary PKA reporter zebrafish. **K** The representative FRET ratio image of ciliary AKAR2-CR in *sx1002*, which were treated with FSK, or injected with cPKA, dnPKA, and Nphp3N-PKI, respectively. **L** Statistical analysis of the ciliary FRET efficiency in *sx1002* in response to FSK, cPKA, dnPKA, and Nphp3N-PKI (*n*_uninjected_ = 18 cilia, *n*_FSK_ = 30 cilia, *n*_cPKA_ = 40 cilia, *n*_dnPKA_ = 24 cilia, *n*_Nphp3N-PKI_ = 16 cilia). **M** Statistical analysis of the ciliary FRET efficiency in *sx1002* treated with gradient FSK (*n* = 30 cilia)

The AKAR2-CR biosensor is a FRET-based PKA activity probe developed to monitor PKA activity in cells [45, 46]. It is composed of a fusion protein consisting of a phosphopeptide binding domain, a Forkhead-associated domain (FHA1), and a consensus region of PKA substrates sandwiched between a donor fluorophore (Clover) and an acceptor fluorophore (mRuby2). In the absence of PKA activity, AKAR2-CR maintains a straight structure such that the Clover and the mRuby2 fluorophores are separated, leading to low FRET efficiency. When the central substrate is phosphorylated by PKA-C, AKAR2-CR undergoes a conformational change that brings the two fluorophores into sufficient proximity to enable efficient FRET. Thus, AKAR2-CR can accurately report PKA activity within cells (Fig. 1A).

To develop a specific PKA biosensor of PC, we fused Nphp3N, which has been shown to be a specific and efficient ciliary targeting peptide (CTP), to the N terminus of AKAR2-CR. When introduced into NIH3T3 cells, the Nphp3N-AKAR2-CR fusion protein was normally expressed and specifically localized to PC (Fig. 1B, C), as indicated by the overlap of Clover and mRuby2 signals with acetylated tubulin, a cilia marker (Fig. 1B). Meanwhile, quantitative comparison revealed no significant difference in ciliary length between cells expressing Nphp3N-AKAR2-CR and wild-type cells (Fig. 1D). To verify whether Nphp3N-AKAR2-CR can sense PKA activity in PC of NIH3T3 cells, we first treated transfected cells with forskolin (FSK). The results showed that the FRET efficiency in PC increased by approximately 9% (Fig. 1E, F). Subsequently, we used 8-Br-cAMP, a membrane-permeable cAMP analog that can directly activate PKA without the need to activate ACs. After stimulation with 10 µM 8-Br-cAMP for 30 min, Nphp3N-AKAR2 expressed in PC of NIH3T3 cells exhibited a 2% increase in FRET efficiency, indicating that elevated cAMP levels specifically trigger the activation of ciliary PKA (Fig. 1E, F). In addition, we used the PKA inhibitory peptide PKIα to specifically interfere with PKA activity. To specifically deliver PKI to cilium, we similarly adopted the Nphp3N targeting peptide fusion strategy. Immunofluorescence staining experiments demonstrated that Nphp3N-PKI was specifically expressed in cilia (Fig. 1G). Detection of cotransfected cells revealed that the FRET efficiency in PC decreased by approximately 2.5% (Fig. 1F). Collectively, these results indicate that the Nphp3N-AKAR2-CR biosensor can effectively report increases in PKA activity in PC of NIH3T3 cells.

The ease of visualization of early embryonic stages in the zebrafish together with robust readouts for cilia and HH pathway activity [47–50] makes it a good model for investigating the role of PKA in HH signaling in vivo. To expand our ciliary PKA biosensor to organism level, the expression and PKA biosensor activity of Nphp3N-AKAR2-CR has been examined. Transient expression of the Nphp3N-AKAR2-CR in zebrafish embryos showed that it localizes specifically in PC as in NIH3T3 cells (Supplementary Fig. S1A). However, the levels of expression varied between individual cells. To overcome this variability, we generated a stable transgenic line ubiquitously expressing Nphp3N-AKAR2-CR throughout embryos under the control of the *β-actin* promoter (Fig. 1J) [51], designated as Tg (*β-actin*::*nphp3N-AKAR2-CR*)^sx1002^ (*sx1002* hereafter). The *sx1002* fish developed normally and were fertile. In nearly all examined ciliated cell types in *sx1002* embryos, both Clover and mRuby2 signals were detected simultaneously within PC (Fig. 1H), suggesting that the Nphp3N-AKAR2-CR fusion protein stably and persistently localizes to PC. In *sx1002* transgenic embryos, no obvious defects in ciliary structure or distribution were observed. Quantitative analysis confirmed that ciliary length in transgenic embryos was not significantly different from that in wild-type controls (Fig. 1I). Furthermore, IFT88, a key component responsible for intraflagellar transport, was properly localized within cilia of *sx1002* embryos expressing Nphp3N-AKAR2-CR (Supplementary Fig. S1B). Together, these findings demonstrate that Nphp3N-AKAR2-CR expression does not affect the structural and functional integrity of PC.

To determine whether changes in PKA activity can be detected in the *sx1002* embryos, embryos were treated with FSK, and relative FRET efficiency was assayed. Consistent with results in NIH3T3 cells, FSK treatment resulted in a marked increase in FRET efficiency within PC of *sx1002* embryos (Fig. 1K, L). These results indicate that the novel ciliary PKA biosensor we have developed can report increased PKA activity in PC of cells and whole organisms. To further test the sensitivity of the Nphp3N-AKAR2-CR biosensor, we evaluated its responsiveness to a gradient of FSK concentrations in *sx1002* embryos. As shown in Fig. 1M, FRET efficiency increased progressively with increasing FSK concentrations, with a subtle yet discernible change observed even at 1 µM FSK. This indicates that the ciliary PKA biosensor exhibits high sensitivity in detecting minute alterations of PKA within PC. Notably, although all tested FSK concentrations increased FRET efficiency in the PC, only 200 µM FSK treatment, resulting in a ~ 7% increase in FRET efficiency, fully phenocopied the HH pathway inhibition induced by cyclopamine (CyA), as revealed by the absence of both Prox1a^+^ and En2a^+^ muscle cells in treated embryos (Supplementary Fig. S2). By contrast, 1 µM and 10 µM FSK elicited modest (~ 1–3%) increases in ciliary FRET efficiency without significantly altering HH pathway activity.

**Fig. 2:**
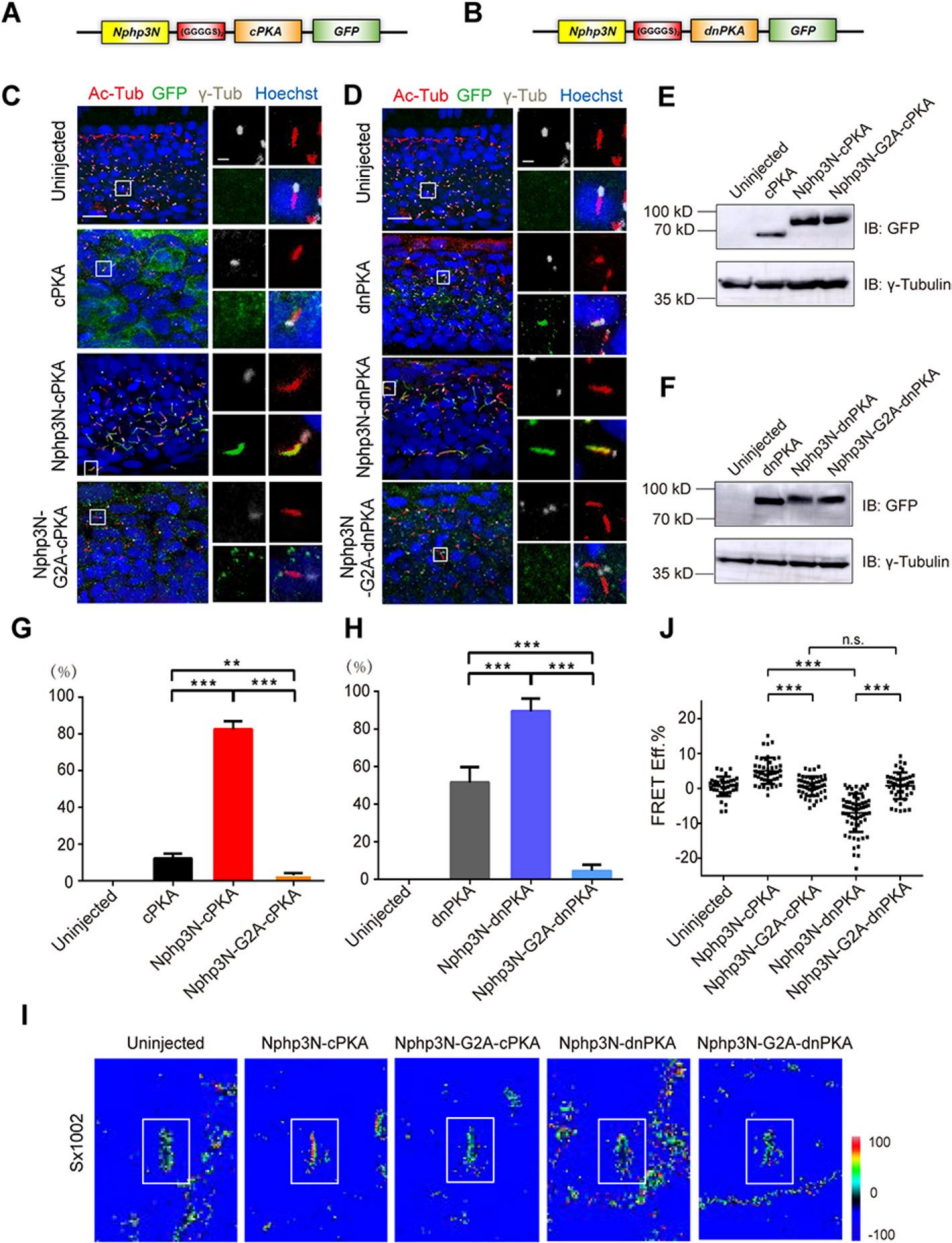
Nphp3N efficiently targets cPKA and dnPKA to the PC. **A**, **B** Schematic diagram of the linker-modified versions of the *Nphp3N-cPKA/dnPKA-eGFP* vector. **C**, **D** Subcellular localization of cPKA/dnPKA-eGFP, Nphp3N(WT/G2A)-cPKA/dnPKA-eGFP in embryos at 18 hpf, as indicated by eGFP. The cilia were labeled by Ac-Tub, the basal body by γ-tubulin, and the nuclei by Hoechst. Frames on the right panel indicates cilia depicted in the insets, with the basal body on upper left, cilia on upper right, indicated cPKAs/dnPKAs on lower left, and merge images on lower right. Scale bars, 10 μm for each left panel and 2.5 μm for the right panel. **E**, **F** Immunoblot of lysates from 18hpf zebrafish embryo expressing indicated GFP-tagged forms of cPKA/dnPKA and Nphp3N(WT/G2A)-cPKAs/dnPKAs. The γ-tubulin was used as loading control. **G** and **H** Quantification of ciliary colocalization from experiments presented in **C** and **D** (*n* = 60 cilia in 3 embryos). **I** The FRET ratio image of ciliary AKAR2-CR in *sx1002* expressing Nphp3N(WT/G2A)-cPKAs/dnPKAs. **J** Statistical analysis of the ciliary FRET efficiency in *sx1002* expressing Nphp3N(WT/G2A)-cPKAs/dnPKAs (*n*_uninjected_ = 37 cilia, *n*_Nphp3N_-_cPKA_ = 46 cilia, *n*_Nphp3N-G2A-cPKA_ = 49 cilia, *n*_Nphp3N_-_dnPKA_ = 54 cilia, *n*_Nphp3N-G2A-cPKA_ = 46 cilia)

Next, we asked whether genetic manipulation of PKA activity can be detected by the ciliary PKA biosensor. Injection of dominant-negative PKA (dnPKA) mRNA or Nphp3N-PKI mRNA into newly fertilized zebrafish eggs resulted in a significant decrease in FRET efficiency within PC of the resulting embryos compared with control embryos (Fig. 1K, L). Consistent with observations in NIH3T3 cells, Nphp3N-PKI localizes to PC (Supplementary Fig. S3A) and functionally exerts moderate activation of the HH signaling pathway(Supplementary Fig. S3B, C). Unexpectedly, however, and contrary to the effects of FSK described above, no significant change of FRET was observed in embryos injected with constitutively active PKA (cPKA) mRNA (Fig. 1K, L). We hypothesized that this could be attributed to the inability of the injected cPKA to localize to PC. To test this, the subcellular localization of cPKA and dnPKA was visualized by expressing fluorescently tagged versions of each protein (cPKA-eGFP and dnPKA-eGFP) in zebrafish embryos. IF staining of 18hpf embryos revealed that cPKA-eGFP was mainly localized in cytoplasm, whereas the dnPKA-eGFP was observed both in cytoplasm and PC (Fig. 2C, D). These results indicate that our biosensor is sensitive to PKA activity specifically in PC.

### Specific delivery of functional PKA to the primary cilia

Given the observed divergence in the subcellular localization of exogenous PKA subunits, we sought to further test the cilia specificity of PKA activity, by targeting both cPKA and dnPKA to PC using Nphp3N CTP. To avoid structural constraints and misfolding of the two fused functional polypeptides, a hydrophilic flexible linker peptide, (GGGGS)_2_, was inserted to increase the distance between Nphp3N CTP and the PKA functional domain, thereby enhancing the proper folding of each domain (Fig. 2A, B). The Nphp3N-cPKA and dnPKA fusions were expressed normally in 18-hpf embryos (Fig. 2E, F) and both localized specifically to PC, with localization efficiency of 82.5% and 89.5%, respectively (Fig. 2C, D, G, H). Nphp3N was also capable of targeting cPKA/dnPKA to cilia in NIH3T3 cells (Supplementary Fig. S4). The glycine of the second residue of Nphp3N is critical for its ciliary targeting function, and the G2A mutation impairs its ciliary localization [32, 52]. Consistently, the Nphp3N-G2A-cPKA and dnPKA fusion proteins were observed to localize only in cytoplasm and not in the shaft of PC (Fig. 2C, D, G, H), thus providing an effective means of comparing cilia- versus cyto-targeted PKA function.

To investigate the activity of ciliary localized Nphp3N-cPKA/dnPKA, mRNAs for either construct were injected into embryos of the *sx1002*. As expected, the cilia-cPKA and dnPKA showed an increase or decrease, respectively, in the FRET efficiency of ciliary AKAR2 (Fig. 2I, J). By contrast, no change of FRET was detected following injection of the cyto-cPKA/dnPKA mRNAs compared with the control group (Fig. 2I, J). Taken together, these results indicate that the newly established ciliary PKA biosensor transgenic line *sx1002* can effectively report changes in PKA activity within PC.

### PKA acts in PC to regulate HH pathway activity

**Fig. 3:**
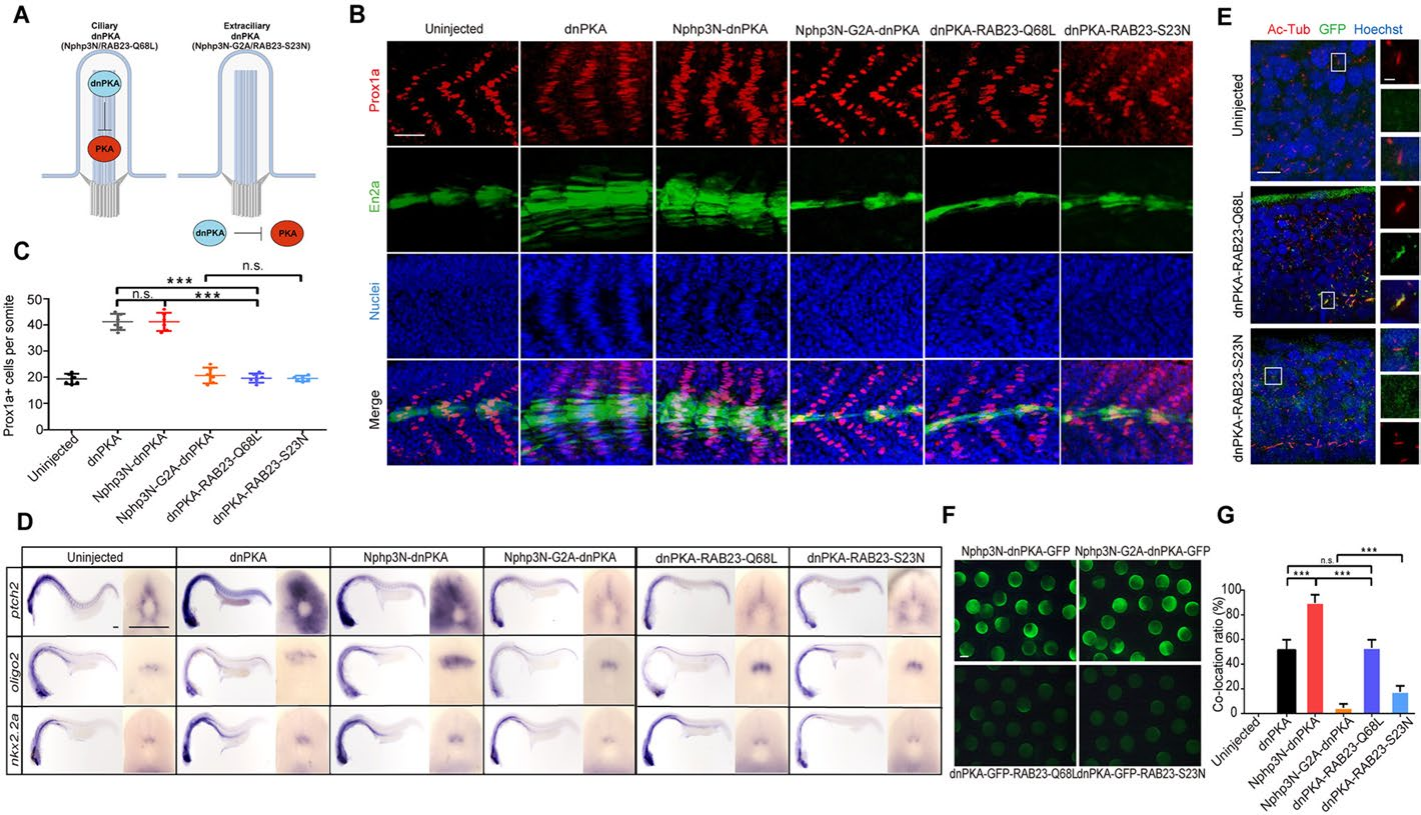
Nphp3N specifically drove dnPKA to PC and efficiently upregulates the HH pathway. **A** Schematic diagram of dnPKA at distinct subcellular locations in this assay. **B** The activity of HH pathway was indicated by expression of Prox1a and En2a:eGFP (En2a) in the embryos of *sx1005* when overexpressing Nphp3N (WT/G2A)-dnPKA and RAB23 (Q68L/S23N)-dnPKA, respectively. The nuclei were labeled by Hoechst. Scale bar, 50 μm. **C** Quantification of Prox1a^+^ cells from experiments presented in **B** (*n* = 6 somites from three embryos). **D** In situ hybridization of *ptch2*, *nkx2.2a*, and *olig2* on the 24-hpf embryos expressing different dnPKAs. Each panel showed a full view of the embryo on the left and a cross-sectional view of a somite on the right (*n* = 3 for each sample). Scale bars, 100 μm. **E** Subcellular localization of the dnPKA-eGFP-Rab23 Q68L/S23N in embryos at 18 hpf, as indicated by eGFP. The cilia were labeled by Ac-Tub, and the nuclei by Hoechst. Frames on the right panel indicate cilia depicted in the insets, with cilia on the top, indicated cPKAs/dnPKAs in the middle, and merge images at the bottom. Scale bars, 10 μm for the left panel and 2.5 μm for the right panel. **F** Transient expression of the indicated Nphp3N (WT/G2A)-dnPKA and RAB23 (Q68L/S23N)-dnPKA in zebrafish embryos at 6 hpf were indicated by eGFP. Scale bars, 500 μm. **G** Quantification of ciliary colocalization from experiments presented in Fig. 2D, E (*n* = 60 cilia in three embryos)

Previously, Truong et al. employed RAB23 variants to target dnPKA to the interior and exterior of cilia and concluded that only ciliary dnPKA could specifically upregulate the HH pathway in zebrafish embryos [53]. However, the activation of HH pathway achieved by this targeted dnPKA was much weaker than that resulting from expression of untargeted dnPKA, as assayed by the expansion of Prox1a and En2a expression in skeletal muscle cells. To investigate this disparity further, we introduced our Nphp3N(G2A), dnPKA constructs into embryos of a newly established transgenic line, *sx1005* (see “Materials and methods”), carrying a previously described *En2a::GFP* reporter construct [34]. In contrast to RAB23-Q68L-targeted cilia-dnPKA, Nphp3N-dnPKA significantly increased the number of En2a/Prox1a double-positive muscle cells, consistent with the previously described hyperactivation of HH pathway effected by injecting dnPKA [11, 48], whereas Nphp3N(G2A)-dnPKA had virtually no effect on HH pathway activity (Fig. 3B, C). Consistently, only Nphp3N-dnPKA induced ectopic upregulation of the Hh pathway target genes, such as *ptch2*, *oligo2*, and *nkx2.2* as shown by in situ hybridization, indicating that cilia-dnPKA mediated by Nphp3N specifically activates the HH pathway, significantly more effectively than the cilia-dnPKA delivered via RAB23-Q68L (Fig. 3D).

We surmised that this reflects the low expression and/or ciliary targeting efficiency of RAB23-Q68L. Accordingly, we compared the efficiency of the two ciliary delivery systems: the RAB23-Q68L-dnPKA and RAB23-S23N-dnPKA constructs were generated, and their expression and subcellular localization were examined by mRNA microinjection into zebrafish embryos. The dnPKA-eGFP fused with RAB23 variants was expressed at much lower levels than the same protein fused with Nphp3N and its variants at 6 h post injection (hpi) and later stages (Fig. 3F). Similarly, the efficiency of ciliary localization of the dnPKA-eGFP fused to RAB23-Q68L was much lower than that of the same protein fused to Nphp3N (Fig. 3E, G).

**Fig. 4:**
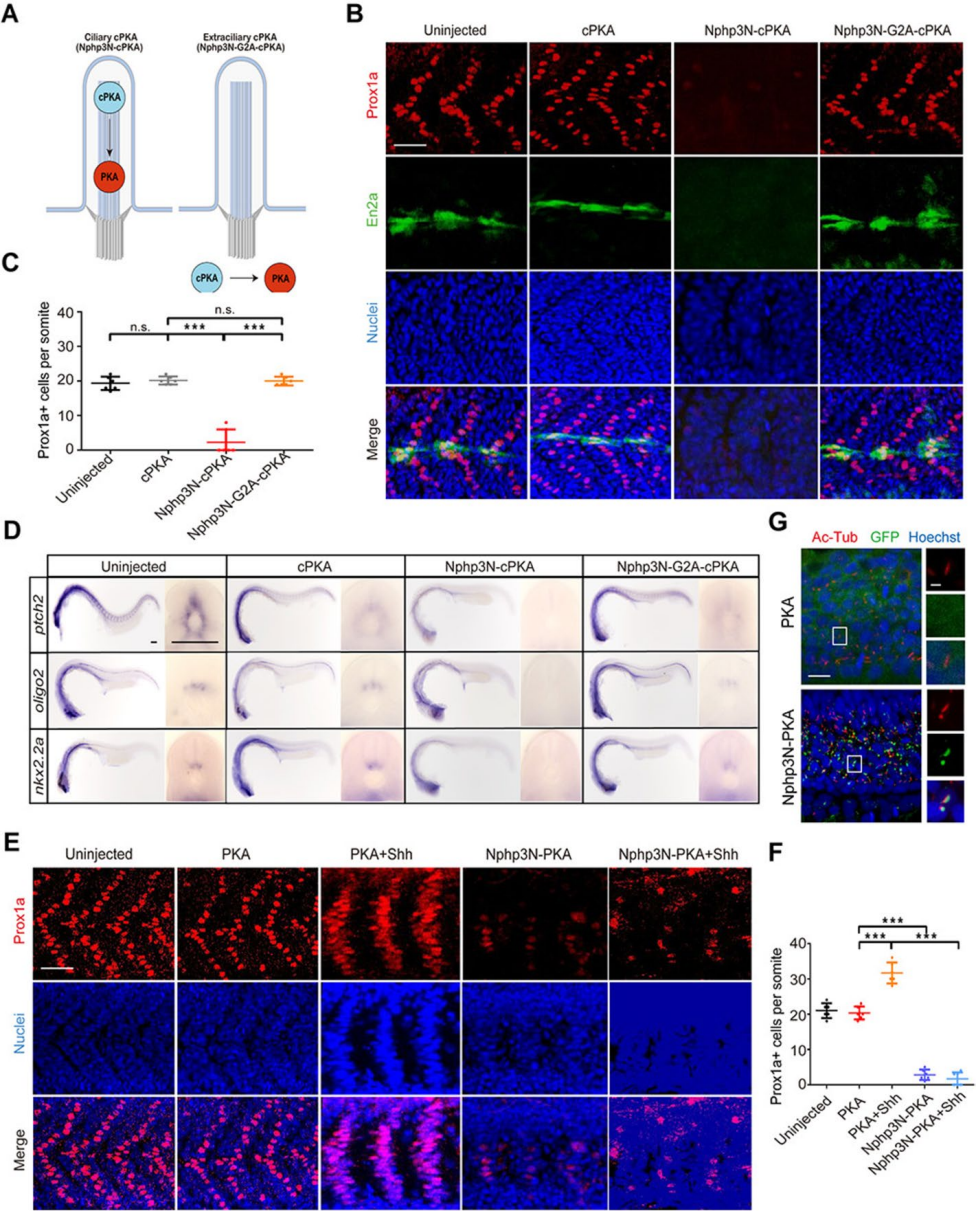
Only cilia-targeted cPKA can modulate HH activity. **A** Schematic diagram of cPKA at distinct subcellular locations in this assay. **B** The activity of HH pathway was indicated by expression of Prox1a and En2a:eGFP (En2a) in the embryos of *sx1005* expressing indicated Nphp3N (WT/G2A)-cPKA. The nuclei were labeled by Hoechst. Scale bar, 50 μm. **C** Quantification of Prox1a^+^ cells from experiments presented in **B** (*n* = 6 somites from three embryos). **D** In situ hybridization of *ptch2*, *nkx2.2a*, and *olig2* on the 24-hpf embryos expressing different cPKAs. Each panel showed a full view of the embryo on the left and a cross-sectional view of a somite on the right (*n* = 3 for each sample). Scale bars, 100 μm. **E** The activity of HH pathway was indicated by expression of Prox1a in the embryos of *sx1005* expressing PKA, PKA + SHH, Nphp3N-PKA, or Nphp3N-PKA + SHH. The nuclei were labeled by Hoechst. Scale bar, 50 μm. **F** Quantification of Prox1a^+^ cells from experiments presented in **E** (*n* = 6 somites in 3 embryos). **G** Subcellular localization of the PKA and Nphp3N-PKA in embryos at 18 hpf, as indicated by eGFP. The cilia were labeled by Ac-Tub, and the nuclei by Hoechst in blue. Frames on the right panel indicate cilia depicted in the insets, with the cilia on the top, the indicated PKA in the middle, and merge layer at the bottom. Scale bars, 10 μm and 2.5 μm (inset)

Although previous studies have confirmed that the RI subunit of PKA is localized to PC [29], the ciliary localization of PKA-C under normal physiological conditions remains unclear [23, 53]. To investigate whether ciliary localization of PKA-C is sufficient for modulation of HH pathway activity, we used the Nphp3N signal peptide to express cPKA specifically in the PC of the *sx1005* transgenic embryos. Injection of mRNA encoding such cilia-targeted cPKA resulted in a significant decrease of En2a^+^ and Prox1a^+^ muscle cells in *sx1005* embryos (Fig. 4B, C). Consistently, the expression of *ptch2*, *oligo2*, and *nkx2.2* was reduced in these embryos (Fig. 4D). By contrast, embryos injected with either untargeted cPKA or the cyto-cPKA showed normal expression of HH-pathway target genes and similar numbers of the En2a^+^ and Prox1a^+^ muscle cells to those in wild-type embryos (Fig. 4B–D). Taken with the finding that the untagged cPKA localizes mainly to cytoplasm (Fig. 2C), this indicates that the HH signaling pathway is inhibited only by PKA activity in PC. We also generated a RAB23-tagged form of cPKA. Consistent with our findings with the RAB23-dnPKA, RAB23-cPKA exerted no significant effect on HH signaling. This is most likely due to its low expression level and suboptimal ciliary targeting efficiency, resulting in minimal impact on PKA activity within the PC (Supplementary Fig. S5).

The failure to detect PKA-C in PC by immunofluorescence under physiological conditions suggests that the low level of PKA-C must be strictly controlled (Fig. 4G). If so, increasing the levels of PKA-C in PC should have a similar effect as the cilia-cPKA. To test this hypothesis, Nphp3N-PKA-C mRNA was injected into embryos. Like the cilia-cPKA, the protein localized to PC (Fig. 4G) and efficiently suppressed HH activity, as shown by the depletion of both Prox1a^+^ and En2a^+^ muscle cells in the myotome (Fig. 4E, F). In contrast, the untagged PKA-C, which was only observed in cytoplasm, had no effect on HH activity (Fig. 4E, F).

### Cilia-targeted PKA is essential even in the absence of PC

In vertebrates, the dependence of HH signaling upon PC is reflected by disruption of HH pathway activity caused by PC aberrations in mouse and zebrafish embryos [54–56]. Our findings imply that HH pathway activity is efficiently suppressed by a basal level of PKA-C activity within PC. Accordingly, loss of PC integrity might lead to suppression of pathway activity being mediated, albeit less efficiently, by cytoplasmic PKA. To investigate the regulation of the HH pathway by ciliary PKA under conditions of ciliary dysfunction, the PC was disrupted in zebrafish embryos by depleting both maternal and zygotic Kif3a, one of the critical factors for primary ciliogenesis [57–60]. As previously shown [60], *MZkif3a* embryos show a reduction in number of PCs in neural tube and otic vesicle cells (Supplementary Fig. S6). Since this mutant line was selected from transgenic strain by both red and green fluorescence [the offspring of Tg(*kop::cas9-p2a-egfp-UTRnanos3*) and Tg(*3* × *sgRNA-kif3a-mcherry*)], to avoid interference from overlapping fluorescent signals, the following staining of Prox1a^+^ and En2a^+^ cells had to be separately detected.

**Fig. 5:**
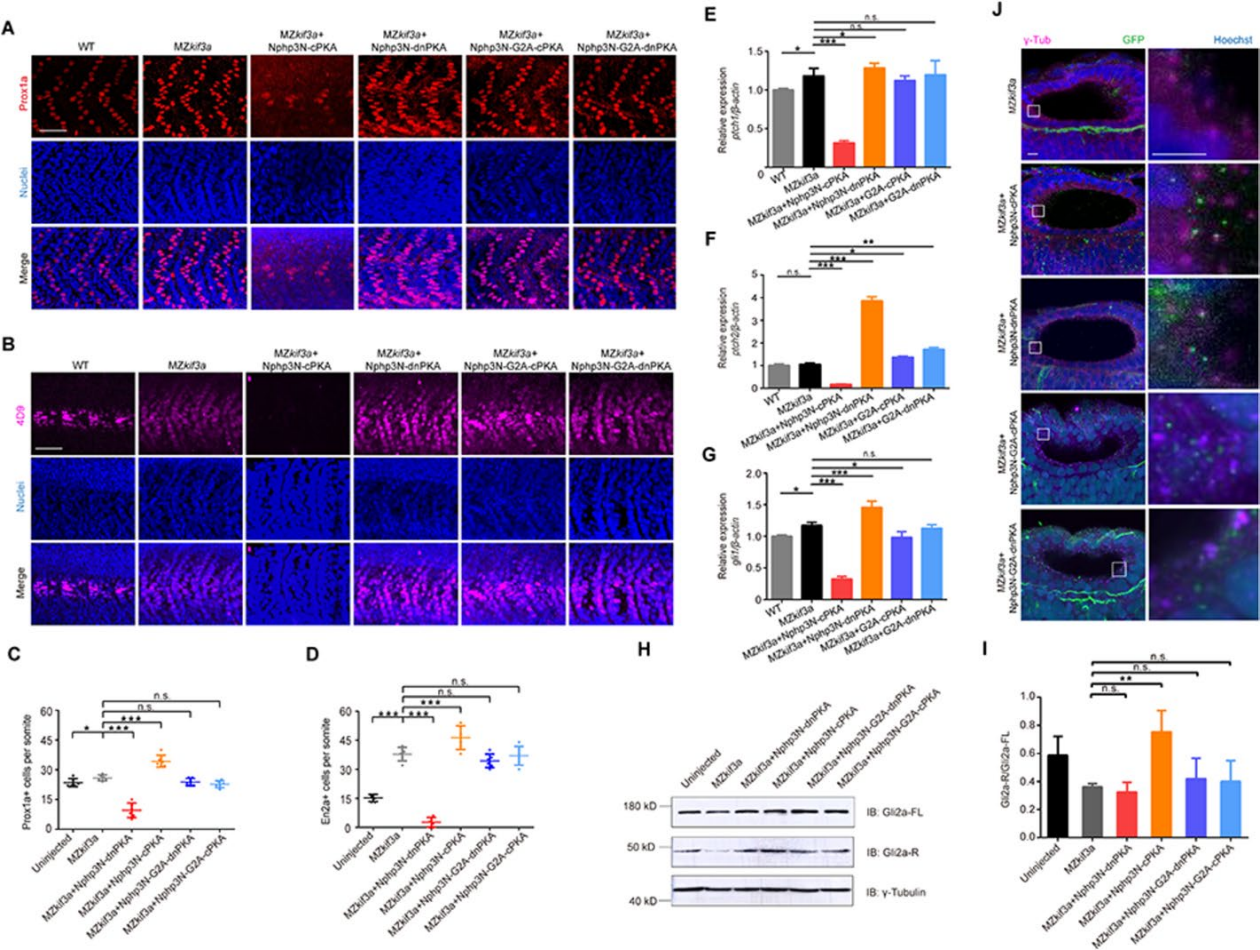
Even in absence of PC, only ciliary PKA can specifically regulates HH pathway. **A** Immunofluorescence staining images of Prox1a in MZ*kif3a* mutants expressing Nphp3N (WT/G2A)-cPKA/dnPKA. **B** Immunofluorescence staining images of En2a in MZ*kif3a* mutants expressing Nphp3N (WT/G2A)-cPKA/dnPKA. **C** Quantification of Prox1a^+^ cells from experiments presented in **A** (*n* = 6 somites from three embryos). **D** Quantification of En2a^+^ cells from experiments presented in **C** (*n* = 6 somites from three embryos). **E**–**G** *ptch1*, *ptch2* and *gli1* expression assay for MZ*kif3a* mutants expressing Nphp3N (WT/G2A)-cPKA/dnPKA at 24 hpf. Data represent the mean ± SD (*n* = 3). **H** Gli2a processing analysis by western blot analysis in MZ*kif3a* mutants expressing Nphp3N (WT/G2A)-cPKA/dnPKA at 24 hpf. Gli2a-FL indicates full-length form of GLi2a and Gli2a-R indicates truncated repressor form of Gli2a. The γ-tubulin was used as loading control. **I** Quantification of the Gli2a-R:FL ratio in **H**. Error bars represent standard deviation obtained from three independent western blots. **J** Subcellular localization of the Nphp3N(WT/G2A)-cPKA/dnPKA in MZ*kif3a* at 24 hpf, as indicated by eGFP. The basal bodies were labeled by gamma tubulin, and the nuclei by Hoechst. The right panel shows the basal bodies and PKA variants in the framework. Scale bars, 10 μm and 2.5 μm (inset)

Compared with wild-type embryos, the number of Prox1a^+^ muscle cells in *MZkif3a* mutants increased slightly, while the number of En2a^+^ muscle cells significantly increased, a phenotype typical of mild upregulation of the HH pathway in cilia defective mutants (Fig. 5A, B). Injection of cyto-cPKA showed little effect in *MZkif3a* mutants (Fig. 5A, B). In contrast, injection of cilia-cPKA into *MZkif3a* mutants resulted in a dramatic reduction in the number of En2a^+^ muscle cells, similar to that observed in wild-type embryos injected with cilia-cPKA (Fig. 5D). Consistently, the number of Prox1a^+^ cells was also greatly reduced (Fig. 5C). These findings indicate that cilia-cPKA inhibits HH pathway activity even in the absence of PC, albeit with a slightly attenuated effect compared to identically treated wild-type embryos. Conversely, injection of cilia-dnPKA into *MZkif3a* mutants led to an increase in Prox1a^+^ and En2a^+^ muscle cells (Fig. 5C, D), comparable to that observed in wild-type embryos, whereas cyto-dnPKA had little effect (Fig. 5A, B). Meanwhile, we tested the transcription levels of HH target genes (*gli1*, *ptch1*, and *ptch2*) in each group under cilia-deficient conditions by quantitative RT-PCR. The results showed that only Nphp3N-tagged cPKA/dnPKA significantly decreased or increased the transcription of *gli1*, *ptch1*, and *ptch2* (Fig. 5E–G). In addition, we assessed the Gli2a processing by western blot. Consistently, Nphp3N-cPKA in cilia significantly increased the Gli2a-R/Gli2a-FL ratio, indicating enhanced proteolytic processing of Gli2a (Fig. 5H, I). However, Nphp3N-dnPKA did not further reduce Gli2a processing in *MZkif3a* mutants (Fig. 5H, I). Similarly, injection of Nphp3N-G2A-cPKA/dnPKA into *MZkif3a* mutant embryos did not result in significant changes in Gli2a processing compared with that in *MZkif3a* (Fig. 5H, I). These results indicate that only ciliary PKA, but not cytoplasmic PKA, can significantly regulate HH pathway activity even in the absence of PC. Notably, neither the ciliary cPKA nor the ciliary dnPKA can fully deplete or increase the numbers of Prox1a^+^ cells in the absence of PC as they do in wild-type embryos, indicating that the regulatory role of PKA in the HH pathway is optimally PC-dependent. Furthermore, subcellular localization analysis revealed that, in *MZkif3a* mutant, cilia-cPKA/dnPKA tended to aggregate in puncta close to the basal body, while the cyto-cPKA/dnPKA displayed ubiquitous cytoplasmic expression (Fig. 5J). This suggests that, in the absence of cilia, ciliary-targeted cPKA and dnPKA may function within a cilia-related compartment near the basal body and that such subcellular localization is critical for PKA-mediated regulation of the HH pathway.

### Overexpression of SMO fails to fully activate the HH pathway through complete inhibition of PKA activity

**Fig. 6:**
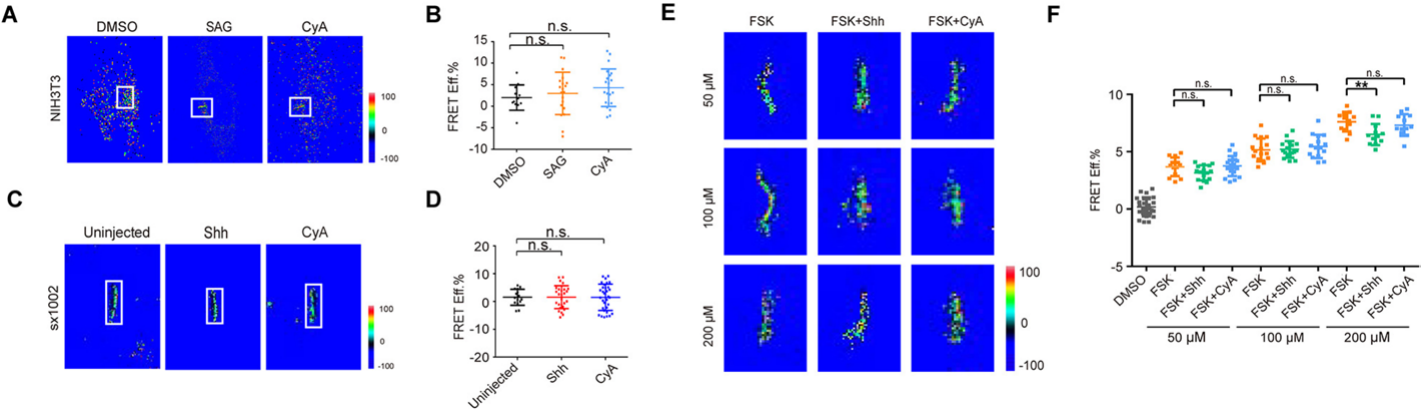
Perturbation of the Hedgehog pathway at the level of SMO has little effect on ciliary PKA activity. **A** The FRET ratio image of ciliary AKAR2-CR in NIH3T3 cells treated with DMSO, 1 μM SAG, and 5 μM cyclopamine (CyA), respectively. **B** Statistical analysis of the ciliary FRET efficiency in NIH3T3 cells in response to DMSO, SAG, and CyA, respectively (*n*_DMSO_ = 12 cilia, *n*_SAG_ = 20 cilia, *n*_CyA_ = 22 cilia). **C** The FRET ratio image of ciliary AKAR2-CR in *sx1002* injected with *shh* mRNA or treated with CyA. **D** Statistical analysis of the ciliary FRET efficiency in *sx1002* treated with *shh* and CyA (*n*_uninjected_ = 18 cilia, *n*_shh_ = 33 cilia, *n*_cyA_ = 31 cilia). **E** The FRET ratio images of ciliary AKAR2-CR in *sx1002* injected with *shh* mRNA or treated with CyA followed by FSK. **F** Statistical analysis of the ciliary FRET efficiency in *sx1002* treated with *shh* and CyA followed by FSK (*n*_DMSO_ = 25 cilia, *n*_50μM FSK_ = 13 cilia, *n*_50μM FSK+shh_ = 16 cilia, *n*_50μM FSK+CyA_ = 19 cilia, *n*_100μM FSK_ = 16 cilia, *n*_100μM FSK+shh_ = 17 cilia, *n*_100μM FSK+CyA_ = 15 cilia, *n*_200μM FSK_ = 12 cilia, *n*_200μM FSK+shh_ = 12 cilia, *n*_200μM FSK+CyA_ = 12 cilia)

Having established that HH pathway activity is modulated specifically by PKA localized to PC, we next investigated the effect of HH pathway activation on PKA. First, NIH3T3 cells transiently expressing Nphp3N-AKAR2-CR were treated with the SMO agonist SAG or the antagonist CyA. Unexpectedly, although both drugs significantly altered HH pathway activity as indicated by changes in the expression of the HH pathway target genes *gli1* and *ptch1* in transfected cells (Supplementary Fig. S7A), we failed to detect any change in FRET efficiency within cilia following either treatment (Fig. 6A, B). Next, we assayed the ciliary PKA activity in *sx1002* zebrafish embryos injected with *shh* mRNA or treated with CyA. As previously reported [48], these treatments resulted in an increase or decrease, respectively, of the number of Prox1a^+^ muscle cells (Supplementary Fig. S7B, C). However, no significant change in PKA activity in PC was detected (Fig. 6C, D). Furthermore, we found that embryos, whether subjected to *shh* mRNA injection to upregulate the HH pathway or treated with CyA to fully suppress it, exhibited comparable responsiveness to FSK treatment as untreated controls. FSK treatment consistently altered ciliary PKA activity in all groups. Notably, FRET efficiency analysis revealed that the efficiency of FSK-induced PKA activation was nearly identical across all conditions (Fig. 6E, F). Only a slight decrease (less than 1%) in ciliary PKA activity was detected under the condition of *shh* mRNA injection combined with 200 µM FSK (Fig. 6E, F). These results suggest that hyperactivation HH pathway does not sequester the ciliary PKA catalytic subunit, as ciliary PKA activity can still be activated by FSK.

**Fig. 7:**
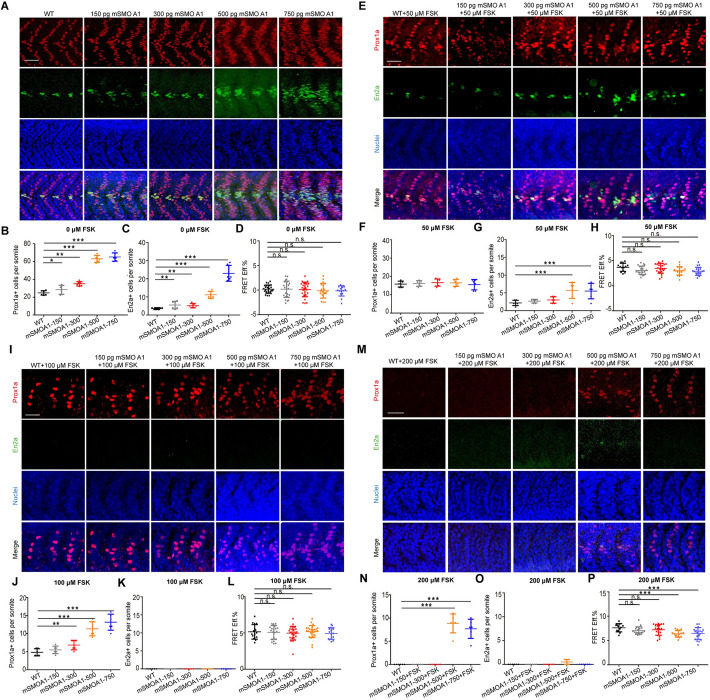
SMO may not control HH pathway activity exclusively by modulation of PKA. **A**, **E**, **I**, **M** Immunofluorescence staining images of Prox1a and En2a in wild-type and those injected with a concentration gradient of mSMOA1 under treatment with varying concentrations of FSK. **A**, 0 μM FSK. **E**, 50 μM FSK. **I**, 100 μM FSK. **M**, 200 μM FSK. The nuclei were labeled by Hoechst. Scale bar, 50 μm. **B**, **F**, **J**, **N** Quantification of Prox1a^+^ cells from experiments presented in **A**, **E**, **I**, and **M** (*n* = 6 somites from three embryos). **C**, **G**, **K**, **O** Quantification of En2a^+^ cells from experiments presented in **A**, **E**, **I**, **M** (*n* = 6 somites from three embryos). **D**, **H**, **L**, **P** Statistical analysis of the ciliary FRET efficiency in *sx1002* injected with a concentration gradient of mSMOA1 under treatment with varying concentrations of FSK. Each group included approximately 25 cilia from three distinct embryos

These findings stand in contrast to the proposal that activated SMO drives the response to HH signals by directly sequestering PKA-C within PC via its PKI-like domain [23, 61, 62]. If this were the case, activated SMO and the ciliary PKA should show a stoichiometric binding relationship upon HH pathway activation. To test this hypothesis, we performed a dose–response titration assay of the constitutively active SMO variant (SMOA1) [63] against FSK in zebrafish embryos. First, we analyzed muscle cell differentiation phenotypes in zebrafish embryos treated with a range of FSK concentrations and SMOA1. As shown in Supplementary Fig. S2, 10 µM FSK induced a very slight reduction in Prox1a^+^ muscle cell number, whereas 100 µM FSK significantly inhibited the HH pathway, as manifested by a loss of En2a^+^ and a marked decrease in Prox1a^+^ muscle cells. At 200 µM, FSK exerted a CyA-like effect, eliminating HH-dependent cell types completely. This result indicates that, at this concentration range, FSK (and by extension, PKA) and the HH pathway activity display a negative linear relationship (Supplementary Fig. S2). Thus, we selected a 50–200 µM range of FSK for subsequent titration experiments. Parallel concentration testing of SMOA1 revealed that, at doses below 500 pg, the number of Prox1a^+^ and En2a^+^ cells increased in a dose-dependent manner (Fig. 7A). However, SMOA1 expression within this range is insufficient to fully activate the HH pathway, while at doses of 500 pg or higher, a robust increase in Prox1a^+^ and En2a^+^ muscle cells was observed, with number of double-positive cells peaking at 750 pg (Fig. 7B, C), indicating excessive activation of the HH pathway under these conditions. Embryos injected with 1000 pg of SMOA1 showed severe deformities, most likely reflecting the toxicity of high concentrations of mRNA (data not shown). Accordingly, we used a range of 150–750 pg for the titration experiments.

Treatment with 200 µM FSK resulted in complete suppression of double-positive cells in all SMOA1 dose groups compared with the untreated control group (Fig. 7M). When the dose of SMOA1 exceeded 500 pg, a few Prox1a^+^ muscle cells were present (no more than ten per somite) (Fig. 7N), whereas En2a^+^ muscle cells were completely absent (Fig. 7O). Thus, SMOA1 overexpression failed to reverse the FSK-mediated inhibition of the HH pathway. To rule out the possibility that nonphysiological levels of PKA activity caused by excessively high FSK concentrations prevented SMOA1 from rescuing FSK-induced HH pathway inhibition, we reduced the concentration of FSK while varying the expression dose of SMOA1. Under 100 µM FSK treatment, the HH pathway was submaximally inhibited (Fig. 7I): En2a^+^ muscle cells were undetectable (Fig. 7K), while the number of Prox1a^+^ muscle cells decreased sharply (Fig. 7J). A slight recovery in the number of Prox1a^+^ muscle cells was observed in embryos injected with 500 pg and 750 pg SMOA1 (Fig. 7J), but no rescue of En2a^+^ cells was evident (Fig. 7K). At 50 µM FSK, only a small increase in En2a^+^ cell number was seen in the 500 pg and 750 pg SMOA1 injection groups (Fig. 7E, G), while there was no significant difference in the number of Prox1a^+^ muscle cells among all groups (Fig. 7E, F). Regarding ciliary PKA activity, no significant changes in FRET efficiency were observed with increasing SMOA1 expression levels across all titrated concentrations of FSK. A weakly discernible reduction in ciliary PKA activity induced by SMOA1 overexpression was detected only at 200 µM FSK (Fig. 7D, H, L, P). These results indicate that PKA-mediated inhibition of the HH pathway cannot be fully relieved by SMO activation and perturbations in SMO levels can only modulate PKA activity within a low-threshold range.

## Discussion

PC play important roles in regulating vertebrate HH signal transduction, and PKA activity in PC is considered to be a critical aspect of the requirement for PC in this signaling cascade; however, the precise details of the processes supported by the PC remain unclear. Compartmentalized activity underpins the precise regulation of key cellular processes mediated by PKA [64–66]. Tracking the activity of PKA in living cells enhances our understanding of this regulation. To achieve this, two strategies could be adopted: measuring either cAMP concentration or PKA activity directly. Tools for monitoring cAMP level in PC using Arl13b-H187 or 5HT6-mCherry-cADDis have been reported [30, 31, 67]. Although the major pathway activated by cAMP is PKA-dependent, cAMP can also activate Epac and cyclic nucleotide-gated (CNG) ion channels directly, independently of PKA [68]. Thus, cAMP level is not synonymous with PKA activity.

To monitor PKA activity in PC, Moore et al. previously utilized the ciliary GPCR 5HT_6_ to target another FRET-based PKA sensor, AKAR4, which reported slightly higher PKA activity in cilia compared with cytoplasm [30]. However, this approach suffers from the possibility that as a typical ciliary GPCR 5HT_6_ could itself modulate PKA activity by coupling to G_αs_, thereby contributing to increase in ciliary PKA activity.

To circumvent these limitations, we have developed an improved sensor for monitoring PKA activity in PC, both in tissue culture and in living embryos. The Nphp3N ciliary targeting peptide (CTP) that we used is much smaller than Arl13b or 5HT_6_ and has minimal effect on PC integrity [32]. In addition, the AKAR2-CR, which uses the Clover-mRuby as the FRET pair, shows higher sensitivity and stability than the AKAR4 that uses the Cerulean-cpVenus as the FRET pair [69]. We generated a stable zebrafish transgenic line ubiquitously expressing the Nphp3N-AKAR2-CR fusion protein, enabling monitoring of PKA activity at the individual PC level. A key advantage of this biosensor is its utility for detecting subtle fluctuations in ciliary PKA activity, outperforming previous tools such as cAMP response element-binding protein (CREB)-based biosensors, which are limited to global cellular readouts [70]. This novel biosensor detected a basal PKA activity, as evidenced by reduced FRET efficiency upon dnPKA expression, and dose-dependent increases with FSK treatment. The level must be strictly controlled as we also failed to detect cPKA within the PC when it was overexpressed. Notably, we found that PKA activity within PC remained largely unchanged upon perturbation of the HH pathway at the level of SMO. Furthermore, we demonstrate that only cilia-targeted PKA, and not its cytoplasmic counterpart, modulates HH signaling, even in the absence of intact cilia, where targeted PKA aggregates near the basal body. These findings underscore the compartmentalized nature of PKA function in HH repression and suggest that a low basal level of ciliary PKA maintains the pathway in an OFF state.

Recently, the use of an alternative ciliary PKA biosensor, based on detection of the phosphorylated vasodilator-stimulated phosphoprotein (pVASP) by immunofluorescence staining, in both zebrafish embryos and NIH3T3 cells has been reported [61]. This PKA biosensor displays high sensitivity in cilia, responding to concentrations of FSK as low as 100 nM. However, unlike our FRET-based Nphp3N-AKAR2-CR sensor, the Arl13b-GFP-VASP sensor does not allow real-time monitoring of PKA activity in PC. Moreover, increasing the levels of Arl13b in PC could cause SMO and GLI2 accumulation independent of exogenous SHH ligand [71, 72]. These factors need to be borne in mind when interpreting the differences in findings using the two sensors.

Although the role of PKA in HH signal transduction is well established, whether PKA regulation of the vertebrate HH pathway is absolutely dependent on PC remains controversial. While a weak signal of PKA-C has been detected in PC 6 h after HH pathway activation with the SMO agonist SAG [23], the ciliary localization of PKA under basal conditions has not been reported. Moreover, rather than localization to the PC, PKA has been reported to localize specifically at the basal body, suggesting that this basal body localization of PKA is critical for its role in the HH pathway [73, 74]. To resolve the subcellular site of PKA action, Truong et al. used RAB23 variants to deliver dnPKA to PC and demonstrated that cilia-dnPKA could specifically activate the HH pathway [53]. However, the magnitude of HH activation by ciliary dnPKA in these experiments was modest. Nevertheless, this spatially restricted manipulation strategy provided a proof of concept for dissecting PKA compartment-specific function. Building on this foundation, ciliary-targeted cPKA and dnPKA using Nphp3N with a (GGGGS)_2_ linker achieved high PC localization efficiency (~82.5–89.5%) in contrast to the lower expression and targeting efficiency of the RAB23 variants. This superior Nphp3N/G2A-mediated targeting enabled precise manipulation. Only ciliary cPKA/dnPKA altered FRET in *sx1002* and HH outputs in *sx1005* embryos, depleting or expanding En2a^+^/Prox1a^+^ muscle cells and targets such as *ptch2*, *oligo2*, and *nkx2.2*. Cytoplasmic variants had no effect, underscoring spatial specificity of PKA in the regulation of HH pathway. This aligns with compartmentalized PKA in vertebrates, where ciliary PKA phosphorylates Gli2/3 at the base, promoting repressor forms [75, 76]. Strikingly, in *MZkif3a* mutants lacking most PCs, ciliary-targeted PKA still modulated HH activity, albeit with attenuated effects and aggregated near basal bodies. This mild HH upregulation observed in mutants was suppressed or enhanced only by ciliary targeted cPKA or dnPKA, while cytoplasmic forms had no effect. Basal body localization suggests that a periciliary compartment, possibly the ciliary pocket or transition zone, serves as a signaling nexus when cilia are absent [77]. This extends the established paradigm of ciliary dependence in vertebrate HH signaling, where defects mildly activate the pathway, to reveal the adaptive capacity of PKA in sustaining HH pathway regulation under ciliary perturbation. Importantly, despite the absence of detectable PKA-C localization in PC, perturbation of ciliary PKA activity at a certain threshold, as achieved with Nphp3N-cPKA and dnPKA (but not RAB23-cPKA/dnPKA), dramatically changed the HH pathway activity in our assays. This indicates that maintenance of the basal ciliary PKA activity is critical for the ON and OFF states of vertebrate HH pathway. This paradox, undetectable PKA-C yet measurable activity, suggests tight regulation, possibly via A-kinase anchoring proteins (AKAPs) that tether PKA to cilia, ensuring localized phosphorylation without bulk accumulation [78, 79].

While our study confirms that basal ciliary PKA activity is critical for regulating the HH pathway, direct monitoring of PKA activity across distinct HH pathway states remains lacking. When perturbing the HH pathway at the level of SMO, our optimized PKA biosensor showed no change in FRET efficiency in NIH3T3 cells or zebrafish embryos in response to either SAG/Shh or CyA, despite robust modulation of expression of HH target genes. There are at least two possible explanations for these results: first, ciliary PKA activity is not significantly changed upon activation or inhibition at the level of SMO; second, the ciliary PKA biosensor is not sensitive enough to detect subtle PKA change during HH signal transduction. Interestingly, we note that, even with the highly sensitive PKA biosensor Arl13b-GFP-VASP, PKA activity in cells treated with SHH/SAG and 100 nM FSK was nearly equivalent to, or slightly higher than, that in control cells [61]. According to the model proposing that SMO directly sequesters PKA-C in PC during the ON state of HH pathway [23, 62, 70] and thereby blocks FSK-mediated effects, ciliary PKA activity in cells treated with SHH/SAG plus FSK should match that in cells treated with SHH/SAG alone. These data support the first hypothesis that ciliary PKA activity is not significantly changed at least upon modulation of the HH pathway at the level of SMO.

Although it has been shown that SMO directly sequesters ciliary PKA-C upon activation of the HH pathway, whether this sequestration is sufficient to activate the pathway remains unclear. To address this, it is necessary to examine the stoichiometric balance of the interaction between SMO and PKA-C in PC. In zebrafish, we observed that 200 µM FSK completely inhibited the HH pathway, while 100 µM FSK exerted strong inhibition, allowing only a small number of Prox1a^+^ muscle cells to differentiate. In contrast, 50 µM FSK caused only a slight reduction in the number of Prox1a^+^ muscle cells, reflecting a mild inhibitory effect on HH pathway activity. On the basis of these observations, we propose that 50 µM FSK represents a suitable and balanced concentration to overcome SMO-mediated activation. At this FSK concentration, if consistent with the SMO–PKA antagonism model, then overexpression of SMO should decrease PKA activity and thereby activate the HH pathway to a measurable extent. However, our titration experiments revealed that low-dose SMO overexpression was insufficient to rescue the FSK-mediated inhibitory phenotype. Even high-dose SMO overexpression (500–750 pg) only resulted in a modest recovery of pathway activity and failed to fully counteract FSK-mediated modulation of HH output. These results suggest that activation of the HH pathway is not solely attributable to SMO-dependent PKA inhibition but involves additional mechanisms, potentially including altered GLI dynamics or ciliary trafficking processes [73, 80].

One achievement of our work is the establishment of a powerful and specific delivery system of functional proteins, especially cPKA and dnPKA, to PC. The smallest cilium target sequence, the Nphp3N signal peptide, significantly reduced the side effect on cilia caused by the signal peptide itself, and the linker sequence ensured the proper function of the proteins delivered to PC. This delivery system provides an excellent opportunity to specifically and efficiently manipulate PC functions, thereby uncovering the mechanism underlying PC-related cellular processes and signal transduction. Notably, our experiments have revealed the effects of PC-targeted PKA on the HH pathway activity. Aberrant activation of the HH pathway underlies the occurrence and progression of several tumors, including basal cell carcinoma and medulloblastoma as well as gastrointestinal and other tumors [81–84], making it a potential target for precision therapeutics. Currently, most drugs targeting this pathway specifically inhibit SMO, exhibit various off-target effects, and are prone to drug resistance. The specific downregulation of the HH pathway activity achieved by the Nphp3N-cPKA transgene suggests the potential for developing novel HH-targeted therapeutics.

In conclusion, we have established a cilia-specific delivery method that achieves high efficiency without compromising the functionality of the targeted proteins. This strategy effectively mitigates any adverse effects of other tags on the structure and function of primary cilia. Targeting PKA activity specifically in PC revealed the existence of a PKA pool whose activity is constrained within a basal range, a pool critical for maintaining HH signaling pathway homeostasis. Our results provide new insights into the mechanism of vertebrate HH signal transduction, while the newly PC delivery system holds promise for precise delivery of multiple proteins and genome-wide tracking of cellular activity, incorporating both temporal and spatial dimensions as well as dynamic information, to enhance the mapping of signal transduction networks.

## Conclusions

In this study, using an improved PKA sensor (Nphp3N-AKAR2-CR), we found that PKA activity in PC remained largely unchanged upon perturbation of the HH pathway at the level of SMO. Furthermore, we demonstrate that only ciliary-targeted PKA, and not its cytoplasmic counterpart, modulates HH signaling, even in the absence of intact cilia, where targeted PKA aggregates near the basal body. These findings underscore the compartmentalized nature of PKA function in HH repression and suggest that a low basal level of ciliary PKA maintains the pathway in an OFF state. Our study provides a new perspective on the mechanism of vertebrate HH pathway activity by PKA. The tool for delivering functional proteins, including cPKA and dnPKA, to PC will enable future studies of protein function in the PC and open new avenues for developing therapeutics targeting PC-related and HH-pathway-associated human diseases.

## Supplementary Information

Additional file 1.

Additional file 2.

## Data Availability

The data that support the findings of this study are available from the corresponding author upon reasonable request.

## Abbreviations

HH: Hedgehog
PC: Primary cilia
PKA: Protein kinase A
PKA-C: PKA catalytic subunit
SMO: Smoothened
FSK: Forskolin
PKA-R: PKA regulatory subunit
cAMP: Cyclic adenosine monophosphate
AC: Adenylyl cyclase
GPCR: G-protein-coupled receptor
PKI: Protein kinase inhibitor
AKAR2: A-kinase activity reporter
Nphp3N: N-terminal region of zebrafish Nephrocystin-3
FHA1: Forkhead-associated domain
CTP: Ciliary targeting peptide
CyA: Cyclopamine
RAB23: Ras-related protein 23
SMOA1: Constitutively active SMO variant
AKAP: A-kinase anchoring protein
FRET: Förster resonance energy transfer
