# Supplementary material for: Hedgehog pathway activity downstream of Smoothened is regulated specifically by basal ciliary PKA
Source: Cell Mol Biol Lett. 2026 Apr 9;31:91. doi: 10.1186/s11658-026-00915-x (PMC13281347; doi:10.1186/s11658-026-00915-x)
Supplement: Supplementary file 1 — Additional file 1. [file 11658_2026_915_MOESM1_ESM.pdf]

Supplemental Figures

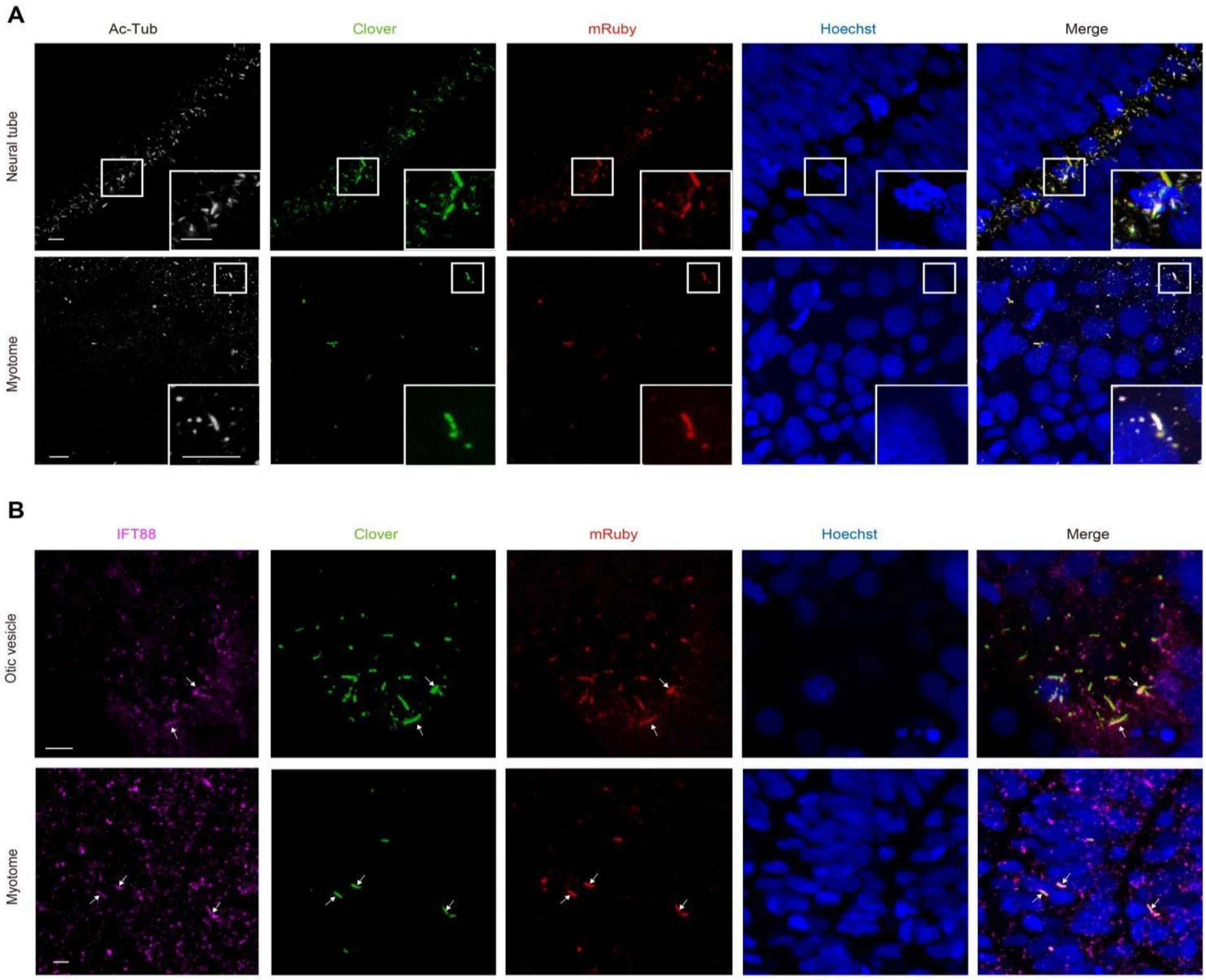

Figure S1. The expression of Nphp3N-AKAR2-CR does not affect the ciliary structure or physiological function.

(A) The transiently expressed zNphp3N-AKAR2 in zebrafish embryos as indicated by Clover in green and mRuby in red was efficiently localized in PC of cells in the neural tube and myotome. Cilia were labeled by Ac-Tub in gray. Each small frame on the lower right corner of the main frame denotes the enlarged site as encircled by white square. The nuclei were labelled by Hoechst in blue. Scale bar, 5 µm. (B) Confocal images show that ciliary transport functions normally in the *sx1002* transgenic zebrafish line. The magenta channel indicates that IFT88 expression is

detectable in both the otic vesicles and neural tubes of the transgenic zebrafish. In the *sx1002*, fluorescence signals are indicated by Clover in green and mRuby in red. The nuclei were labelled by Hoechst in blue. Arrows denote distinct IFT88 signals within the cilia of the transgenic line. Scale bar, 5 μm.

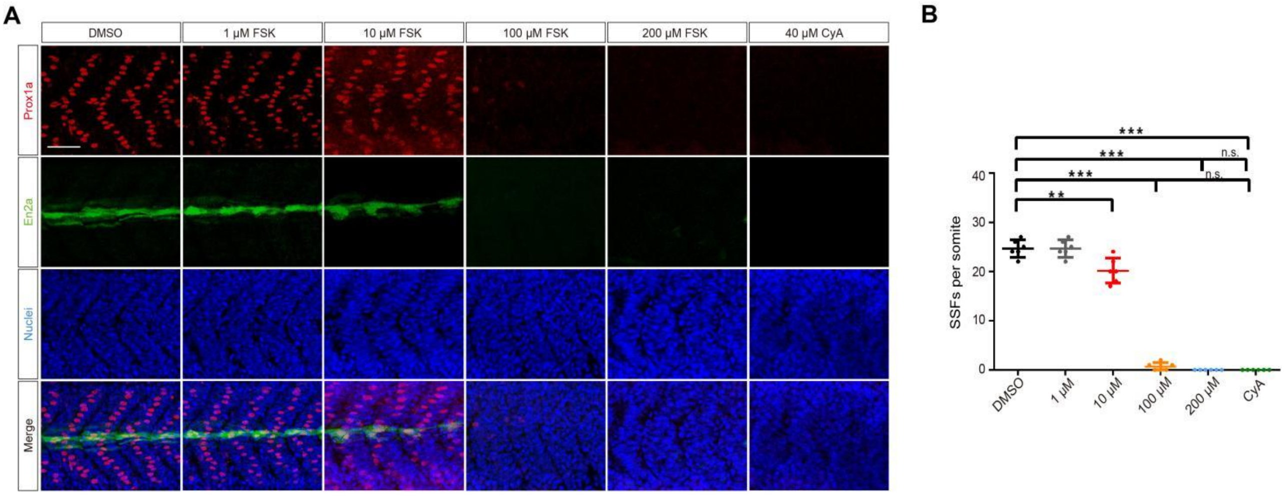

Figure S2. Treating zebrafish embryos with forskolin reduced the expression of Prox1a and En2a in the developing somite.

(A) The activity of HH pathway was indicated by expression of Prox1a in red and En2a:eGFP in green in the embryos of *sx1005*, a reporter of Hedgehog signal transduction. Embryos were treated with increasing doses of FSK and 40 μM CyA from 6 to 24hpf, respectively. The nuclei were labelled by Hoechst in blue. Scale bar, 50 μm. (B) Quantification of Prox1a+ cells from experiments presented in A (n = 6 somites in 3 embryos).

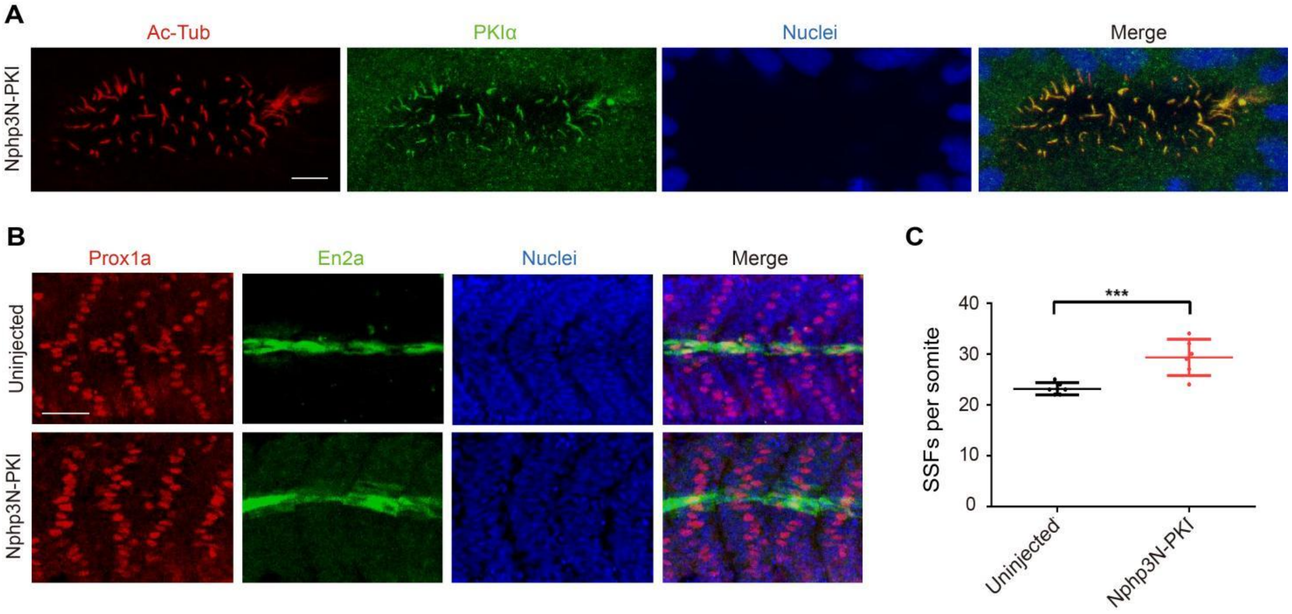

Figure S3. Nphp3N-PKI moderately upregulates the HH pathway.

(A) Representative images showing colocalization of Nphp3N-PKI with PC in the otic vesicle of wild-type zebrafish embryos. Cilia were labeled by Ac-Tub. The nuclei were labelled by Hoechst. Scale bars, 5 μm. (B) The activity of HH pathway was indicated by expression of Prox1a and En2a:eGFP (En2a) in the embryos of *sx1005* when overexpressing Nphp3N-PKI, respectively. The nuclei were labelled by Hoechst. Scale bar, 50 μm. (C) Quantification of Prox1a+ cells from experiments presented in B (*n* = 6 somites from 3 embryos).

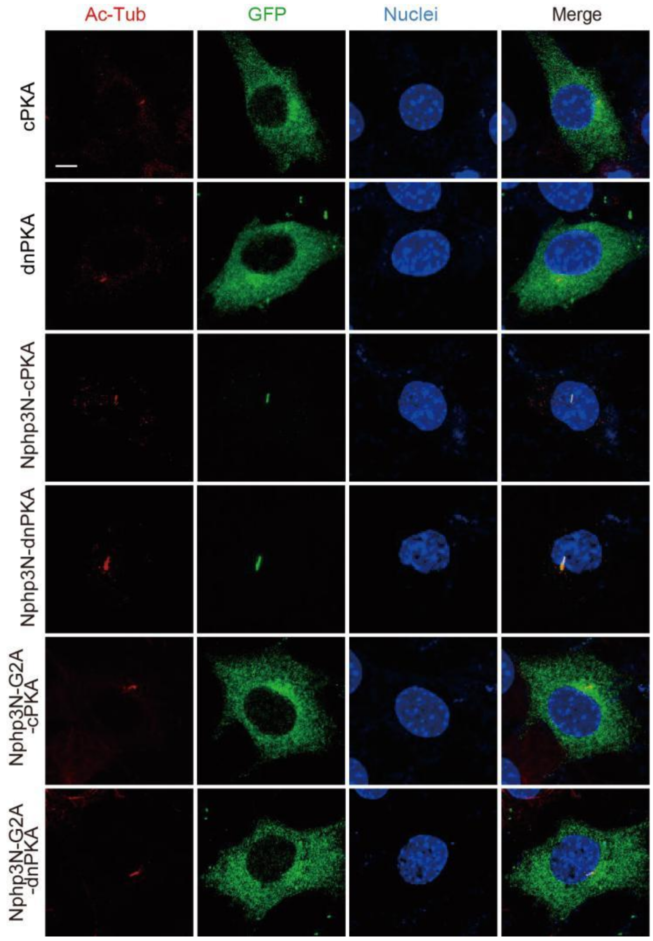

Figure S4. Nphp3N specifically drove cPKA/dnPKA to the PC in NIH3T3 cell.

Subcellular localization of the different forms of cPKA/dnPKA when transiently expressing in NIH3T3 cells, as indicated by eGFP in green. The cilia were labeled by Ac-Tub in red and the nuclei by Hoechst in blue. Scale bars, 5 μm.

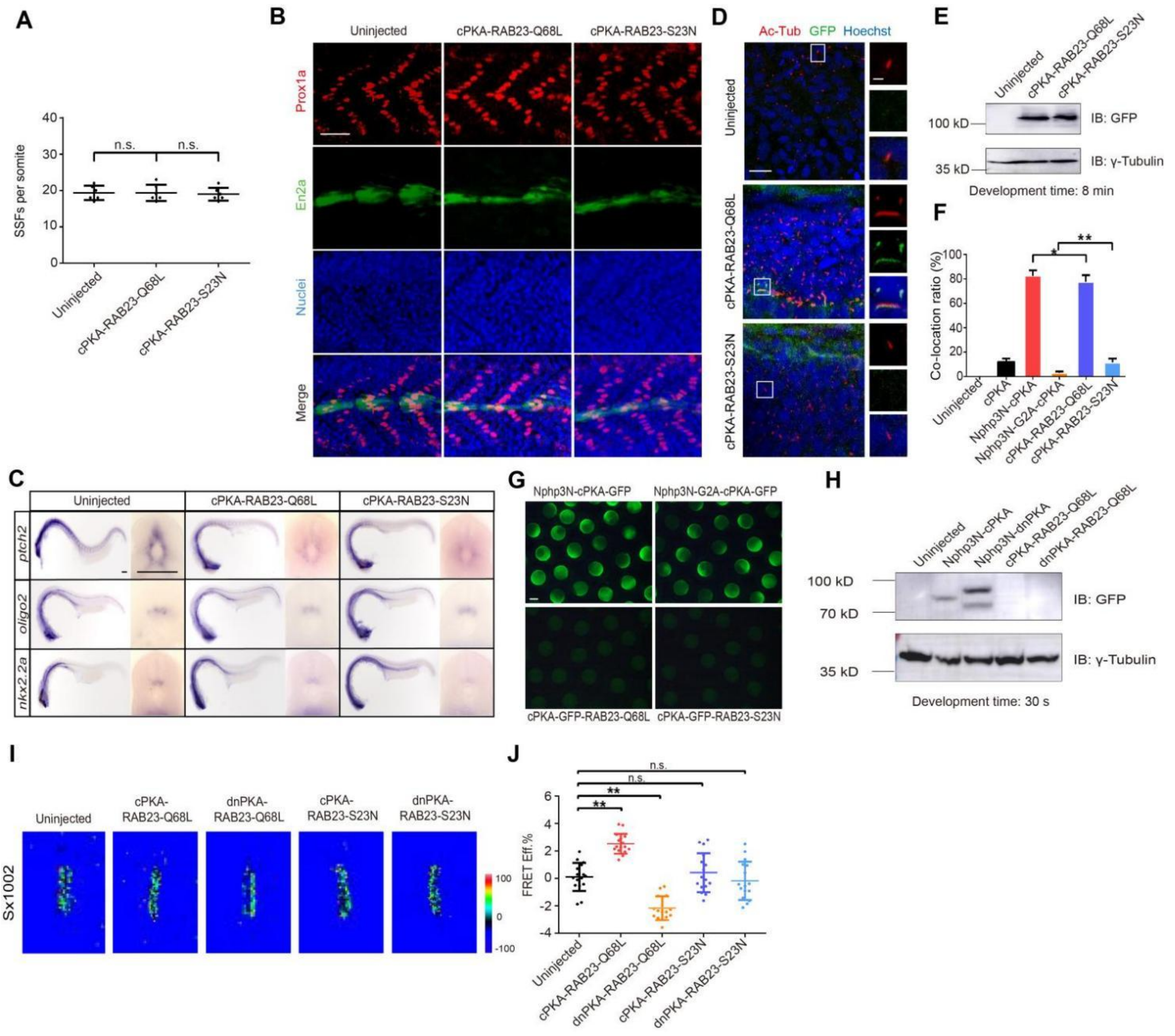

Figure S5. The cPKA driven by the RAB23 variant only weakly regulates the HH pathway.

(A) Quantification of Prox1a+ cells from experiments presented in B (n = 6 somites from 3 embryos). (B) The activity of HH pathway was indicated by expression of Prox1a in red and En2a:eGFP in green in the embryos of *sx1005* expressing indicated RAB23 (Q68L/S23N)-cPKA. The nuclei were labelled by Hoechst in blue. Scale bar, 50 µm. (C) *In situ* hybridization of *ptch2*, *nkx2.2a* and *olig2* on the 24 hpf embryos expressing indicated cPKAs. Each panel showed a full view of the embryo on the left and a cross-sectional view of a somite on the right (n = 3 for each sample). Scale bars, 100 µm. (D) Subcellular localization of the cPKA-eGFP-Rab23 Q68L/S23N

in embryos at 18 hpf, as indicated by eGFP in green. The cilia were labelled by Ac-Tub in red, and the nuclei by Hoechst in blue. Scale bars, 10 μm for the left panel and 2.5 μm for the right panel. (E) Immunoblot of lysates from 18 hpf zebrafish embryo expressing indicated GFP-tagged forms of RAB23-cPKAs. The γ-tubulin was used as loading control. Development time, 8 min. (F) Quantification of ciliary colocalization from experiments presented in Fig 2B and D (n = 60 cilia in 3 embryos). (G) Transient expression of the indicated Nphp3N (WT/G2A)-cPKA and RAB23 (Q68L/S23N)-cPKA in zebrafish embryos at 6 hpf were indicated by eGFP in green. Scale bars, 500 μm. (H) Immunoblot of the indicated fusion proteins in embryos. The γ-tubulin was used as loading control. Development time, 30 s. (I) The FRET ratio image of ciliary AKAR2-CR in *sx1002* expressing RAB23 (Q68L/S23N)-cPKAs/dnPKAs. Representative images of the FRET ratio for each condition in the pseudocolor scale. (J) Statistical analysis of the ciliary FRET efficiency in *sx1002* expressing RAB23 (Q68L/S23N)-cPKAs/dnPKAs.

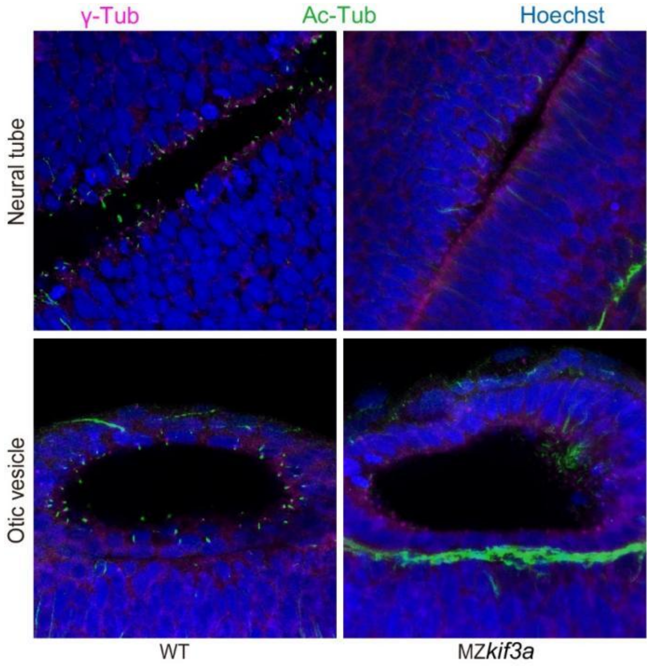

Figure S6. Ciliary are completely absent in MZ*kif3a* mutants.

Cilia were labelled with acetylated tubulin in green and the basal bodies were stained with gamma tubulin in magenta. Nuclei were stained with Hoechst in blue. Scale bar, 10 µm.

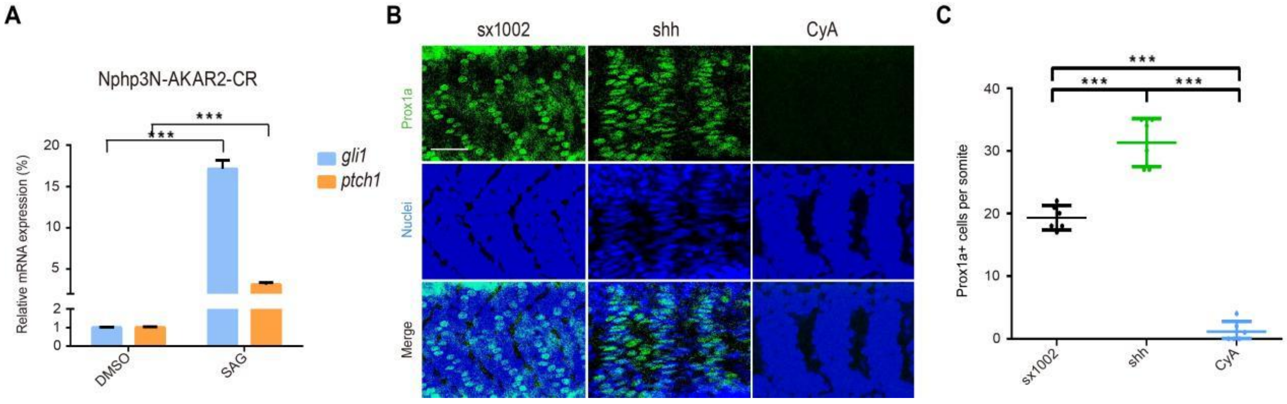

Figure S7. The expression of Nphp3N-AKAR2-CR does not affect the function of HH modulators.

(A) NIH3T3 cells were transfected with Nphp3N-AKAR2-CR, then treated with 1 μM SAG or an equivalent volume of DMSO, respectively. Expression levels of *gli1* and *ptch1* were subsequently quantified. Data represents the mean and ± SD (n = 3). (B) Representative Prox1a (red)/nuclei (blue) immunostainings of *sx1002* embryos at 24 hpf after injected with *shh* mRNA or treated with CyA, respectively. Scale bars, 50 μm. (C) Quantification of Prox1a+ cells from experiments presented in B (*n* = 6 somites from 3 embryos).
