# Supplementary material for: Hedgehog pathway activity downstream of Smoothened is regulated specifically by basal ciliary PKA
Source: Cell Mol Biol Lett. 2026 Apr 9;31:91. doi: 10.1186/s11658-026-00915-x (PMC13281347; doi:10.1186/s11658-026-00915-x)
Supplement: Supplementary file 2 — Additional file 2. [file 11658_2026_915_MOESM2_ESM.pdf]

Supplemental Table

Table S1. FRET experimental conditions for NIH3T3 cell line

Drug	Source	Final concentration	Identifier	Treatment
Forskolin	MedChemExpress	10 µM	HY-15371	Serum-free transfection 24h post-treatment for 30 min
8-Br-cAMP	MedChemExpress	10 µM	HY-12306A	Serum-free transfection 24h post-treatment for 30 min
SAG	MedChemExpress	1 µM	HY-12848	Serum-free transfection 24h post-treatment for 24h
cyclopamine	MedChemExpress	5 µM	4449-51-8	Serum-free transfection 24h post-treatment for 24h

Table S2. List of Primers

Name	Used for	Sequence
Nphp3N-cPKA-F	Cloning	AGGCGCAAGGCATTCAGATTATGGGCAACGCGCCCACCG
pCS2-Nphp3N-R	Cloning	AATCTGAATGCCTTGCGCCTG
pCS2-Nphp3N-GFP-F	Cloning	CCCGGGATGGTGAGCAAGGG
Nphp3N-cPKA-GFP-R	Cloning	CCCTTGCTCACCATCCCGGGGAATTCAGCAAACTCCTTTCC
Nphp3N-dnPKA-F	Cloning	AGGCGCAAGGCATTCAGATTATGGCGTCTGGCAGTATG
Nphp3N-dnPKA-GFP-R	Cloning	CCCTTGCTCACCATCCCGGGGACGGACAGGGACACGAA
prkacaa-F	Cloning	ATAGGATCCCCACCATGGGCAACGCGCCCACCGCCAAG
prkacaa-R	Cloning	ATATCTAGACTAGAATTCAGCAAACTCCTTTC
PKAH89Q-F	Cloning	AACACTGAATGAGAAACGCATCC
PKAH89Q-R	Cloning	TGTTCTATCTGTTTCAGCTTCA
PKAW198R-F	Cloning	AGGACACTGTGTGGCACACCTGA
PKAW198R-R	Cloning	GGTCCTGCCCTTTACACGTTTA
Nphp3N-linker-cPKA-F	Cloning	GCTCAGGCGGCGGCGGCTCAATGGGCAACGCGCCCACCG
pCS2-Nphp3N-linker-R	Cloning	AGCCGCCGCCGCCTGAGCCGCCGCCGCCAATCTGAATGCCTTGCGC
pCS2-eGFP-F	Cloning	CCCGGGATGGTGAGCAAGGG
Nphp3N-linker-dnPKA-F	Cloning	GCTCAGGCGGCGGCGGCTCAATGGCGTCTGGCAGTATG
pCS2-Nphp3N-linker-R	Cloning	AGCCGCCGCCGCCTGAGCCGCCGCCGCCAATCTGAATGCCTTGCGC
linker-cPKA-mCherry-R	Cloning	TCACCATAGATCCACCTCCACCGAATTCAGCAAACTCCTTTCC
linker-mCherry-F	Cloning	GGTGGAGGTGGATCTATGGTGAGCAAGGGCGAGG
linker-dnPKA-mCherry-R	Cloning	TCACCATAGATCCACCTCCACCGACGGACAGGGACACGAAG
mcherry-pCS2-R	Cloning	TACGACTCACTATAGTTCTAGATTACTTGTACAGCTCGTCCATGC
pCS2-F	Cloning	TCTAGAACTATAGTGAGTCGTATT
dnPKA-R	Cloning	GACGGACAGGGACACGAAGC
cPKA-R	Cloning	CCTGGTGTGTGCCATCCTCCTG
Nphp3N-G2A-F	Cloning	CTACGGCATCCTCTCTGGTGAGCCCTGGTGAG
Nphp3N-G2A-R	Cloning	ACCAGAGAGGATGCCGTAGCCATGGTGGG

		GATCCTGCAAA
linker-RAB23-F	Cloning	GGCTCAGGCGGCGGCGGCTCAATGTTGGAGGAAGATATGGAAGT
linker-GFP-R	Cloning	AGCCGCCGCCGCCTGAGCCGCCGCCGCCCTTGTACAGCTCGTCCATGC
pCS2-RAB23-R	Cloning	AATACGACTCACTATAGTTCTAGATTAGGGTACACTACAGCTGCT
RABS23N-F	Cloning	AATAGCATGATTCAGCGCTACTGC
RABS23N-R	Cloning	CTTTCCAACCGCCCCATTCC
RABQ68L-F	Cloning	TAGAGGAGTTTGATGCAATCACG
RABQ68L-R	Cloning	GACCTGCAGTGTCCCATAACA
pCS2-ATGR	Cloning	CATGGTGGCTGGATCCTGCAAAAAG
AKAR2woATGF SmoA1	Cloning	GTGCGGGGTTCTCATCATCATC
mIFT88-F	Cloning	TTTTGCAGGATCCAGCCACCATGATGGAAAATGTTCATCTGGCA
mIFT88-R	Cloning	TTGTAGTCACTTGCAGCTGCTTCTGGGAGCAAGTCATCTCC
Ptch1-exon20- F	qPCR	CATCGGTGACAGGAACAAGC
Ptch1-exon21- R	qPCR	CTCAGGATAAGGGCCAAAGTAG
ptch2- F	qPCR	CCTGGTGTGTGCCATCCTCCTG
ptch2- R	qPCR	TCCATAGCAACGGCTGAACGAG
β-actin-F	qPCR	CTCTTCCAGCCTTCCTTCCT
β-actin-R	qPCR	CACCGATCCAGACGGAGTAT
gli1-F	qPCR	GGAGACGCCTCGTGTTTCT
gli1-R	qPCR	AACCCAACGATGGACTCATGG

Table S3. List of Antibodies

Antibody	Source	Dilution	Identifier	Used for
acetylated α-tubulin	Sigma	1:1000	T7461	Immunofluorescence
γ-tubulin	Sigma	1:1000	T3320	Immunofluorescence and Western Blotting
GFP	ABclonal	1:1000	AE012	Western Blotting
Gli2a-R	GeneTex	1:800	GTX128280	Western Blotting
GFP	Abcam	1:1000	AB13970	Immunofluorescence
Prox1	Abcam	1:1000	AB5475	Immunofluorescence
4D9	Abcam	1:100	AB12454	Immunofluorescence
IFT88	Proteintech	1:200	13967-1-AP	Immunofluorescence
PKIA	Proteintech	1:80	11743-1-AP	Immunofluorescence
Hoechst 33258	BBI Life Science	1:10000	E607329-0010	Immunofluorescence
Alexa Fluor 647 anti-rabbit	ThermoFisher	1:1000	A31573	Immunofluorescence
Alexa Fluor 546 anti-rabbit	ThermoFisher	1:1000	A10040	Immunofluorescence
Alexa Fluor 488 anti-mouse	ThermoFisher	1:1000	A21202	Immunofluorescence
Alexa Fluor 488 anti-chicken	ThermoFisher	1:1000	A11039	Immunofluorescence
